# Biodegradable Harmonophores for Targeted High-Resolution *In Vivo* Tumor Imaging

**DOI:** 10.1021/acsnano.0c10634

**Published:** 2021-02-25

**Authors:** Ali Yasin Sonay, Konstantinos Kalyviotis, Sine Yaganoglu, Aysen Unsal, Martina Konantz, Claire Teulon, Ingo Lieberwirth, Sandro Sieber, Shuai Jiang, Shahed Behzadi, Daniel Crespy, Katharina Landfester, Sylvie Roke, Claudia Lengerke, Periklis Pantazis

**Affiliations:** † Department of Biosystems Science and Engineering (D-BSSE), Eidgenössische Technische Hochschule (ETH) Zurich, 4058 Basel, Switzerland; ‡ Department of Bioengineering, Imperial College London, South Kensington Campus, London SW7 2AZ, U.K.; § Department of Biomedicine, University Hospital Basel and University of Basel, 4031 Basel, Switzerland; ∥ Laboratory for Fundamental BioPhotonics, Institute of Bioengineering, School of Engineering, École Polytechnique Fédérale de Lausanne, 1015 Lausanne, Switzerland; ⊥ Max Planck Institute for Polymer Research, 55128 Mainz, Germany; ○ Division of Pharmaceutical Technology, Department of Pharmaceutical Sciences, University of Basel, 4031 Basel, Switzerland; ∇ Department of Materials Science and Engineering, School of Molecular Science and Engineering, Vidyasirimedhi Institute of Science and Technology (VISTEC), Rayong 21210, Thailand; ¶ Institute of Materials Science and Engineering, School of Engineering, École Polytechnique Fédérale de Lausanne, 1015 Lausanne, Switzerland; ◆ Lausanne Centre for Ultrafast Science, École Polytechnique Fédérale de Lausanne, 1015 Lausanne, Switzerland; % Division of Hematology, University Hospital Basel, 4031 Basel, Switzerland

**Keywords:** self-assembly, ferroelectric, biodegradable, second harmonic generation, imaging

## Abstract

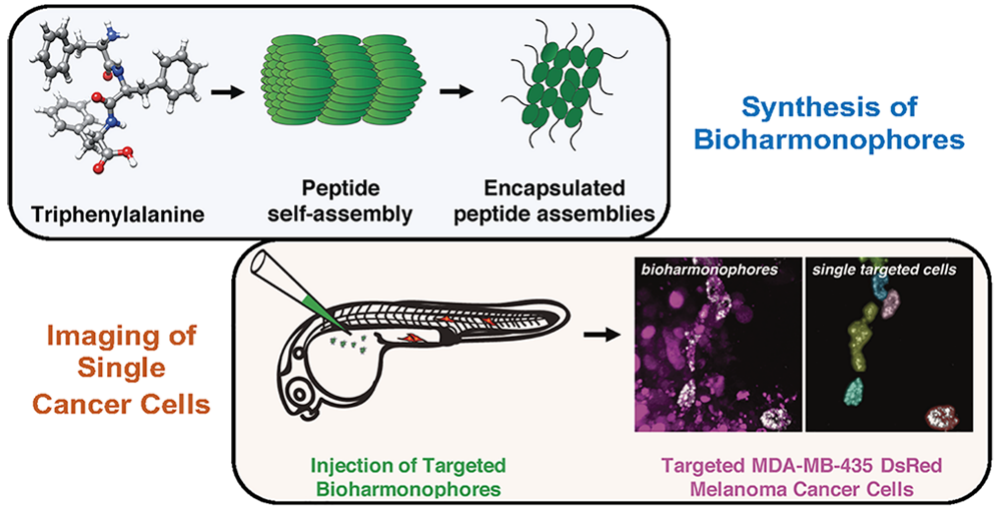

Optical imaging probes have played a major role in detecting and monitoring a variety of diseases. In particular, nonlinear optical imaging probes, such as second harmonic generating (SHG) nanoprobes, hold great promise as clinical contrast agents, as they can be imaged with little background signal and unmatched long-term photostability. As their chemical composition often includes transition metals, the use of inorganic SHG nanoprobes can raise long-term health concerns. Ideally, contrast agents for biomedical applications should be degraded *in vivo* without any long-term toxicological consequences to the organism. Here, we developed biodegradable harmonophores (bioharmonophores) that consist of polymer-encapsulated, self-assembling peptides that generate a strong SHG signal. When functionalized with tumor cell surface markers, these reporters can target single cancer cells with high detection sensitivity in zebrafish embryos *in vivo*. Thus, bioharmonophores will enable an innovative approach to cancer treatment using targeted high-resolution optical imaging for diagnostics and therapy.

Clinical and preclinical imaging holds great potential in mapping disease progression and can provide diagnostic information^1^ that may guide the choice of treatment strategies for disease.^2,3^ Optical techniques using bioluminescent and fluorescent probes have emerged as promising modalities for molecular imaging in disease and therapy due to their ease of use and improved cellular resolution, capable of distinguishing boundaries between malignant and normal tissue.^4^ A key challenge for optical imaging probes and instrumentation, particularly those aimed at eventual clinical applications, is to overcome the problem of autofluorescence, which often result in a low signal-to-noise ratio (SNR).^5^ The relatively poor photostability of most imaging probes poses another challenge to provide reliable and sensitive imaging of tumors.

Previously, we introduced inorganic second harmonic generating (SHG) nanocrystals, SHG nanoprobes,^6^ as a class of imaging probes that can be used for *in vivo* imaging.^7^ Given that SHG imaging employs near-infrared (NIR) incident light for contrast generation, SHG nanoprobes can be utilized for deep tissue imaging.^8,9^ Unlike commonly used fluorescent probes, SHG nanoprobes neither bleach nor blink, and their signal does not saturate with increasing illumination intensity, ensuring high probe sensitivity.^10^ Since their signal profile is very narrow, they can be imaged with high SNR by excluding the broad emission of typical autofluorescence background.^6,11^ Robust functionalization allows targeting to a wide variety of cells and proteins of interest,^12^ allowing these imaging probes to be promising tools for both clinical and preclinical imaging applications.^13^ Despite these advantages, the chemical structure of inorganic SHG nanoprobes makes them stable in the body, which may cause concerns for the long-term health of an organism that has been imaged with these reporters.^14^

To create a foundation for safe SHG nanoprobe-based clinical imaging, we set out to generate a nanoprobe that consists of biodegradable materials, capable of generating sufficient SHG signal that can be detected with high SNR. Our efforts were guided by the observation that peptides with a variable number of amino acid units can self-assemble into large, solid nanostructures of different morphologies and symmetries (Figure 1a).^15^ It has been previously shown that such structures can be ferroelectric and give nonlinear optical contrast such as SHG (Figure 1c).^16,17^ Since these peptides self-assemble in solution in an inherently sequential process, they form nanostructures in the micrometer range that are difficult to control in size and cannot be readily utilized for *in vitro* and *in vivo* imaging. Given that the SHG signal output is dependent on the size of the assembly and significantly decreases at the nanoscale, we set out to develop a synthesis method that would miniaturize these peptides assemblies and control their size into nanoparticles. Importantly, miniaturized peptide assemblies should not lose their crystalline structure and SHG signal, generating imaging agents that can be degraded over time without harming the body.

**Figure 1:**
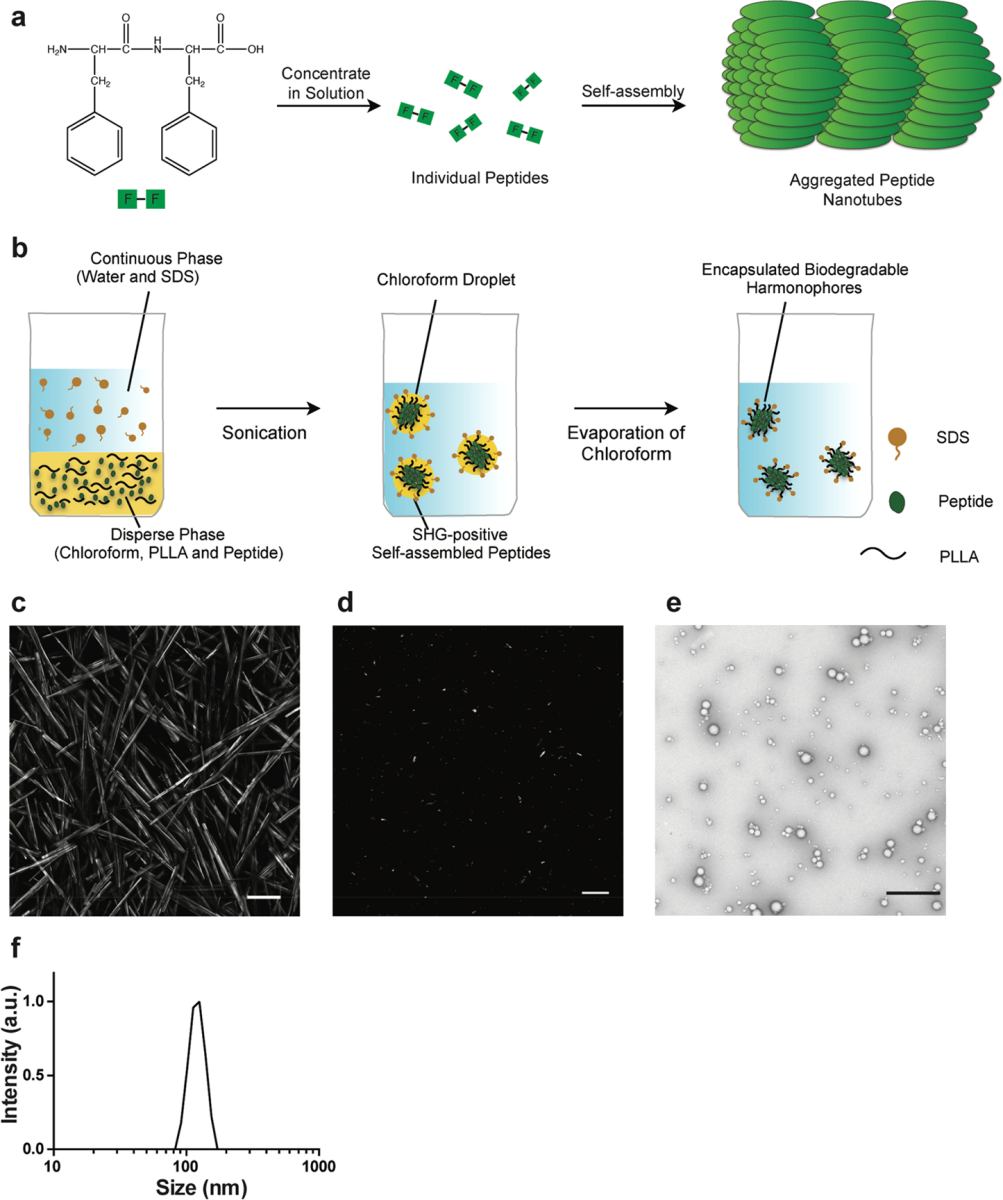
Synthesis and analysis of bioharmonophores. (a) Schematic of the self-assembling reaction of diphenylalanine peptides (FF) into large-scale nanotube structures from a concentrated solution. (b) Schematic of the emulsion–solvent evaporation method for the synthesis of bioharmonophores. Self-assembling peptides are dissolved in chloroform along with biodegradable poly(l-lactic acid) (PLLA) and emulsified with the surfactant sodium dodecyl sulfate (SDS) using sonication, followed by evaporation of chloroform. (c) SHG signal from diphenylalanine peptide nanotubes aggregated on top of the imaging chamber. Peptide nanotubes were illuminated with a 850 nm pulsed laser. Image composite of multiple stitched images. (d) SHG signal from encapsulated triphenylalanine peptide (FFF) bioharmonophores immobilized in 1% low melting agarose illuminated with 850 nm pulsed laser. (e) TEM image of synthesized FFF-based bioharmonophores showing uniform spherical nanoparticles. (f) DLS data showing the size distribution of synthesized bioharmonophores. Scale bar, 100 μm (c); 10 μm (d); 500 nm (e).

## Results and Discussion

### Synthesis of Bioharmonophores

To render these nanostructures suitable for biological applications, we evaluated methods for the encapsulation of self-assembling peptides in order (i) to hinder their macroscopic aggregation by confining their self-assembly in nanodroplets without affecting their ability to generate a strong SHG signal and (ii) to generate a nanoparticle that can be further functionalized without influencing the peptide assembly. To this end, we subjected several peptides that have been reported to self-assemble into complex nanostructures to the emulsion–solvent evaporation method,^18^ a widely used procedure for the fabrication of monolithic and core–shell nanoparticles (Figure 1b, see the Experimental Methods).

We identified three peptides with different self-assembling properties (pentaalanine,^19^ trileucine,^20^ and triphenylalanine^21^) that could generate a detectable SHG signal when encapsulated in the biodegradable polymer (Figure 1d and Supplementary Figure 1). Transmission electron microscopy (TEM) analysis of the predominantly spherical nanoparticles, hereinafter referred to as bioharmonophores, revealed a diameter ranging from 50 to 150 nm (Supplementary Figure 2), which was confirmed by dynamic light scattering (DLS) measurements (Figure 1e,f).

SHG signal from bioharmonophores can stem from (i) the bulk of the self-assembling peptides that form noncentrosymmetric crystalline structures or (ii) the surface of the bioharmonophores where there is no inversion symmetry. To ascertain that the SHG signal originates from the crystalline peptide core, we performed X-ray diffraction (XRD) analysis of bioharmonophores with different peptide contents. In all cases, the peptides showed a high degree of internal order with distinct diffraction patterns associated with their individual crystalline phases and self-assembling behavior (Supplementary Figure 3a–d).

### Optical Characterization of Bioharmonophores

**Figure 2:**
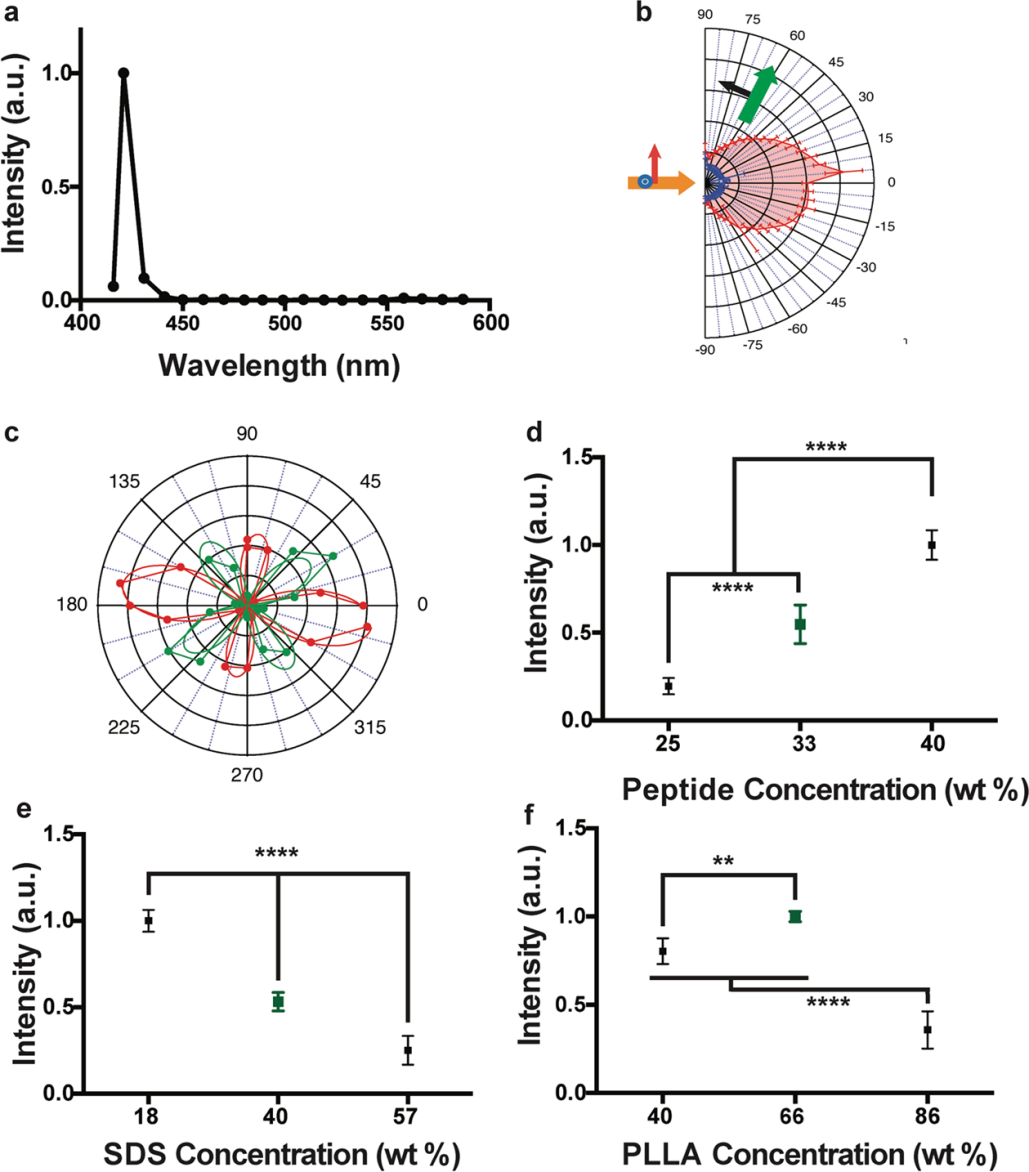
Optical characterization of bioharmonophores and analysis of parameters influencing bioharmonophore formation. (a) Normalized SHG signal spectrum of FFF-based bioharmonophores (signal ranging from 400 to 600 nm) illuminated with 850 nm pulsed laser. The characteristic SHG peak is centered around 425 nm. (b) SHG emission pattern of Triphenylalanine based bioharmonophores. Orange arrow indicates excitation beam direction. Green arrow shows SHG collection direction, which rotates between −90° and +90°. The detected polarization is in the beams plane (P, black arrow). The red pattern shows PPP polarization configuration (excitation and detection polarizations in the plane of the beams), and the blue pattern shows PSS (excitation with a perpendicular polarization). (c) SHG intensity *vs* incident polarization angle for a bioharmonophore, highlighted by the solid white circle in Supplementary Figure 5. Red color shows detection along the X axis while green color shows detection along the Y axis. The experimental curve is a dotted line, the corresponding fitted curve, assuming *C*_*2*_ symmetry, is a solid line. (d) Influence of using different amounts of FFF peptide during bioharmonophore production on the SHG signal intensity. The optimal condition (33 wt %) is marked in green. The use of higher FFF peptide amount leads to aggregates (*n* = 5). (e) Influence of SDS concentration (wt % of disperse phase) on SHG intensity of generated bioharmonophores. The optimal condition (40 wt % SDS) with high bioharmonophore stability and less aggregation is marked in green (*n* = 5). (f) Influence of using different amounts of PLLA during bioharmonophore production on the SHG intensity of the generated bioharmonophores. The optimal condition (66 wt % PLLA) is marked in green (*n* = 5). Mean ± s.d. ****, *P* < 0.0001, **, *P* < 0.005, *, *P* < 0.05 (ordinary one-way ANOVA with Tukey’s multiple comparisons).

Because bioharmonophores based on triphenylalanine (FFF) peptides yielded the strongest SHG signal compared to pentaalanine and trileucine, we subjected these bioharmonophores to detailed optical characterizations. The SHG signal of FFF-based bioharmonophores was spectrally well-defined (Figure 2a) and comparable with inorganic SHG nanoprobes (Supplementary Figure 4). Additionally, the SHG emission patterns of FFF-based bioharmonophores displayed a broad opening: one seemingly isotropic (Figure 2b, PSS polarization, blue data) and the other one displaying one lobe with a width of 60° centered around the forward direction (Figure 2b, PPP polarization, red data). These results indicate that bioharmonophores emit an SHG signal in multiple directions, which can be detected in an epi-detection setup (*i.e.*, illumination and collection of SHG signal using the same objective lens), unlike the predominantly forward-directed SHG signal of large protein arrays, such as collagen and myosin that require a trans-detection approach (*i.e*., illumination and SHG signal traverses the sample to reach the detector).^10^ This difference in detection geometry allows exclusion of a collagen-based SHG signal during imaging, leading to a high SNR bioharmonophore signal that can be distinguished from large-scale collagen arrays even in tissues with high SHG background. Moreover, the presence of a single lobe suggests that the observed SHG signal originates from the bulk^22^ of the bioharmonophores and not from its surface, as described by nonlinear light scattering theory.^22,23^ The high intensity emission in the PPP polarization combination further indicates that a coherent response originates from a noncentrosymmetric crystalline bulk.

Because SHG involves only virtual energy transitions, bioharmonophores did not display blinking and remained stable over extended periods of illumination, and their SHG signal intensity rose quadratically when the laser intensity shone on them was linearly increased (Supplementary Figure 5a,b). The measured polarimetric diagrams (Figure 2c and Supplementary Figure 5c–f) were consistent with the hypothesis that bioharmonophores have a self-assembling peptide core with a monoclinic (*C*2) symmetry. Indeed, the experimental curves were well fitted with the analytical expression calculated for this symmetry (see Supplementary Note 1). Taken together, bioharmonophores have the same photophysical advantages for biomedical imaging applications that have been previously described for inorganic SHG nanoprobes.^6^

To gain insight into the parameters influencing the bioharmonophore stability and signal intensity, we tested several reaction conditions to generate bioharmonophores. Given that the SHG signal originating from bioharmonophores is dependent on the amount of encapsulated peptide, we first tested whether varying the FFF peptide concentration during production would improve the SHG signal intensity of generated bioharmonophores (Figure 2d and Supplementary Figure 6). We found that an amount of 15 mg of FFF (33 wt %) peptide provided an optimal combination of intense SHG signal and bioharmonophore stability. Interestingly, while an FFF peptide amount of 20 mg (40 wt %) increased the overall SHG signal, it also led to bioharmonophore aggregation and decreased colloidal stability. Conversely, 10 mg (25 wt %) of FFF peptide generated little SHG signal.

Because surfactant concentration plays a crucial role in emulsification of chloroform droplets,^18^ we reasoned that altering the surfactant concentration during the preparation of bioharmonophores would have a profound effect on their stability and signal strength (Figure 2e and Supplementary Figure 7). Bioharmonophores emulsified in an aqueous solution with 0.3% sodium dodecyl sulfate (SDS) (40 wt % of dispersed phase) yielded stable bioharmonophores with intense SHG signal, whereas compositions employing 0.1% SDS (18 wt %) yielded aggregated nanoparticles. Increasing the SDS concentration to 0.6% (57 wt %) diminished the SHG signal intensity, suggesting that the bioharmonophore size and hence the number of enclosed peptide molecules within each bioharmonophore is influenced by the concentration of surfactant.

Finally, we varied the polymer quantity that encapsulates and shields peptides from environmental changes and assessed its role in both SHG signal intensity and nanoparticle morphology (Figure 2f and Supplementary Figure 8). We identified that an amount of 30 mg of poly(l-lactic acid) (PLLA) (66 wt %) resulted in an optimal combination of intense SHG signal and bioharmonophore stability. A lower polymer amount of 10 mg (40 wt %) yielded a weaker SHG signal, whereas a higher polymer amount (90 mg, 86 wt %) led to elongated bioharmonophore morphologies. Taken together, we identified optimal experimental conditions to generate bioharmonophores providing a high SNR along with an excellent stability and size distribution for biological applications.

### Biodegradation of Bioharmonophores

**Figure 3:**
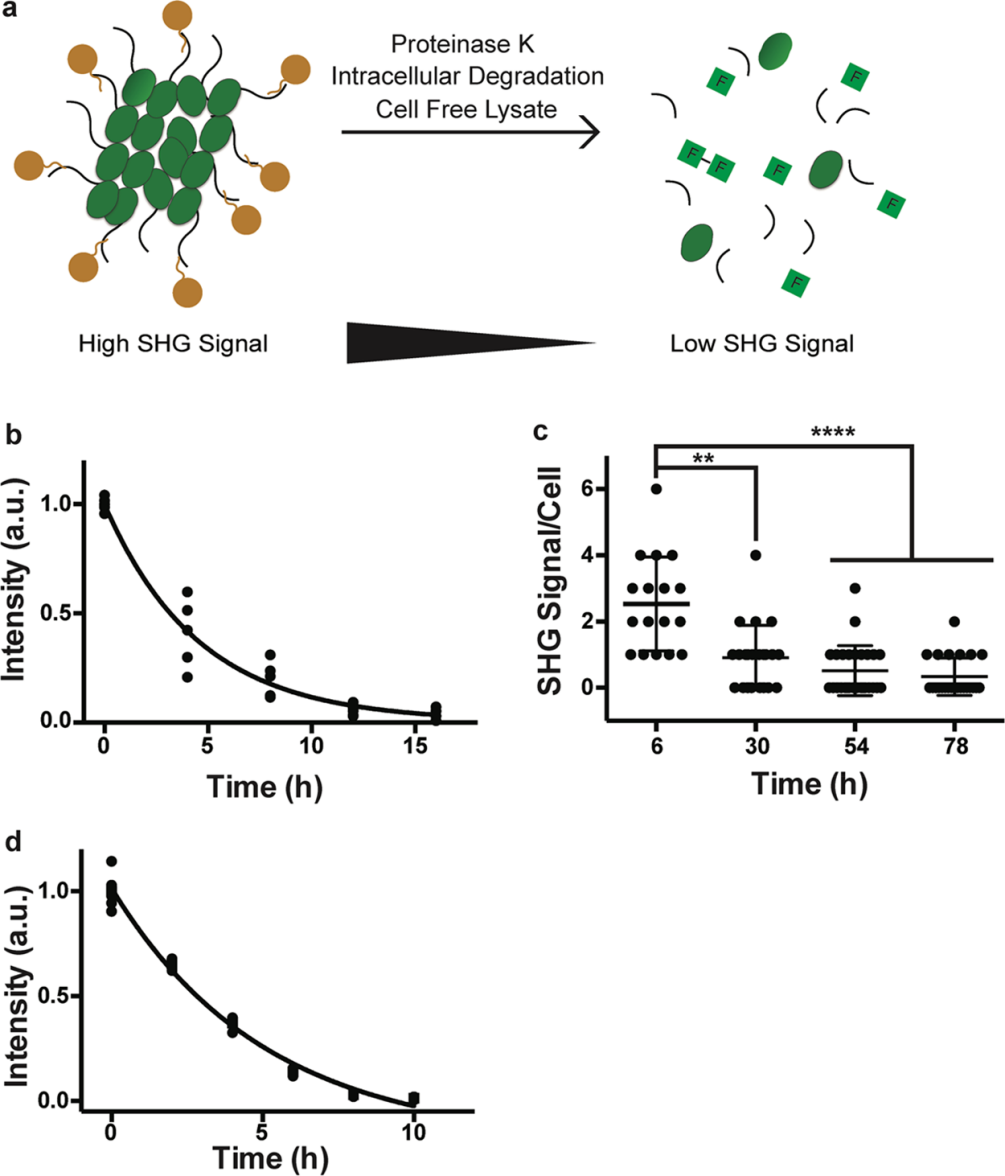
Bioharmonophores can be degraded by proteases, cells, and cell-free lysate systems. (a) Schematic showing different degradation methods utilized to assess biodegradability of the bioharmonophores. (b) Graph displaying the change of SHG signal intensity over time of bioharmonophores incubating with proteinase K (*n* = 5). Mean values of data points were fitted for one-phase exponential decay. (c) Quantification of SHG signal/cell after overnight incubation of Tat-peptide functionalized bioharmonophores over time. SHG signal/cell is significantly reduced 30 h after reseeding. Mean ± s.d. ****, *P* < 0.0001, **, *P* < 0.005, *, *P* < 0.05 (nonparametric Kruskal–Wallis test with Dunn’s post hoc multiple comparison). (d) Graph showing the loss of SHG signal intensity when bioharmonophores are subjected to the cell-free reticulate lysate degradation system (*n* = 5). Mean values of data points were fitted using a one phase exponential decay. Scale bar, 10 μm (c); 10 μm (d).

Clinical imaging probes that are biodegradable provide the significant advantage of being able to be broken down in the body and removed after they have served their function. To demonstrate that bioharmonophores are indeed biodegradable, we utilized the highly effective serine protease, proteinase K, which exhibits a broad cleavage specificity.^24^ We incubated bioharmonophores with a proteinase K concentration that is routinely used for dissolving tissue structures^25^ and probed the extent of degradation by monitoring the SHG signal at different time intervals (Figure 3a). We observed a decrease of SHG signal within 2 h of protease incubation. After 10 h, the SHG signal disappeared and the turbid bioharmonophore suspension became transparent (Figure 3b and Supplementary Figure 9), indicating a successful biodegradation of the bioharmonophore. When characterized by TEM imaging, the structural integrity of the bioharmonophore coat started to disintegrate within 4 h of Proteinase K treatment (Supplementary Figure 10).

To evaluate bioharmonophore degradation under physiological conditions (Figure 3c), we functionalized bioharmonophores with Tat-derived cell penetrating peptides^26^ using bioorthogonal click chemistry (Supplementary Figure 11) and incubated them with a model cancer cell line (see below) overnight. Adherent cells were then detached by trypsinization and centrifuged to remove excess bioharmonophores that did not enter the cancer cells. Following this procedure, cells were reseeded and fixed at specific time periods to monitor bioharmonophore degradation (*i.e.*, the intracellular presence of SHG signal per cell) using nonlinear optical imaging. Thirty hours after cell reseeding, a pronounced decrease of intracellular SHG signal per cell was noticeable (Figure 3c). As bioharmonophores displayed long-term photostability even at low pH values (Supplementary Figure 12), the drop of signal was not due to their potential accumulation in acidic endolysosomal compartments over time. Immunostaining of uptaken bioharmonophores indeed revealed that bright SHG signal still persisted in lysosome-associated membrane protein 2 (LAMP2)-positive endocytic compartments prior to degradation (Supplementary Figure 13). In order to show that bioharmonophores can be degraded using intracellular proteolytic degradation, we tested whether the bioharmonophores could be degraded using a cell-free lysate system based on an established cell-free degradation assay.^27^ We also observed reduced SHG signal, indicating that intracellular enzymatic degradation of bioharmonophores might account for the signal loss (Figure 3d and Supplementary Figure 14). Importantly, bioharmonophores did not exhibit any short-term toxicity *in vitro* and *in vivo* (Supplementary Figure 15) and did not induce protein aggregation (Supplementary Figure 16),^28^ rendering them safe imaging probes.

### *In Vivo* Cancer Imaging

Among various diagnostic applications, bioharmonophores could be ideal imaging probes for single-cell cancer detection due to their high SNR and photostability, which other intravital imaging modalities cannot achieve.^2^ To demonstrate the optical features of bioharmonophores for cancer targeting and imaging, we employed xenograft zebrafish cancer models, which offer speed, cellular resolution, and the ability to perform large numbers of transplants for obtaining valuable information about several cancer types.^29,30^

**Figure 4:**
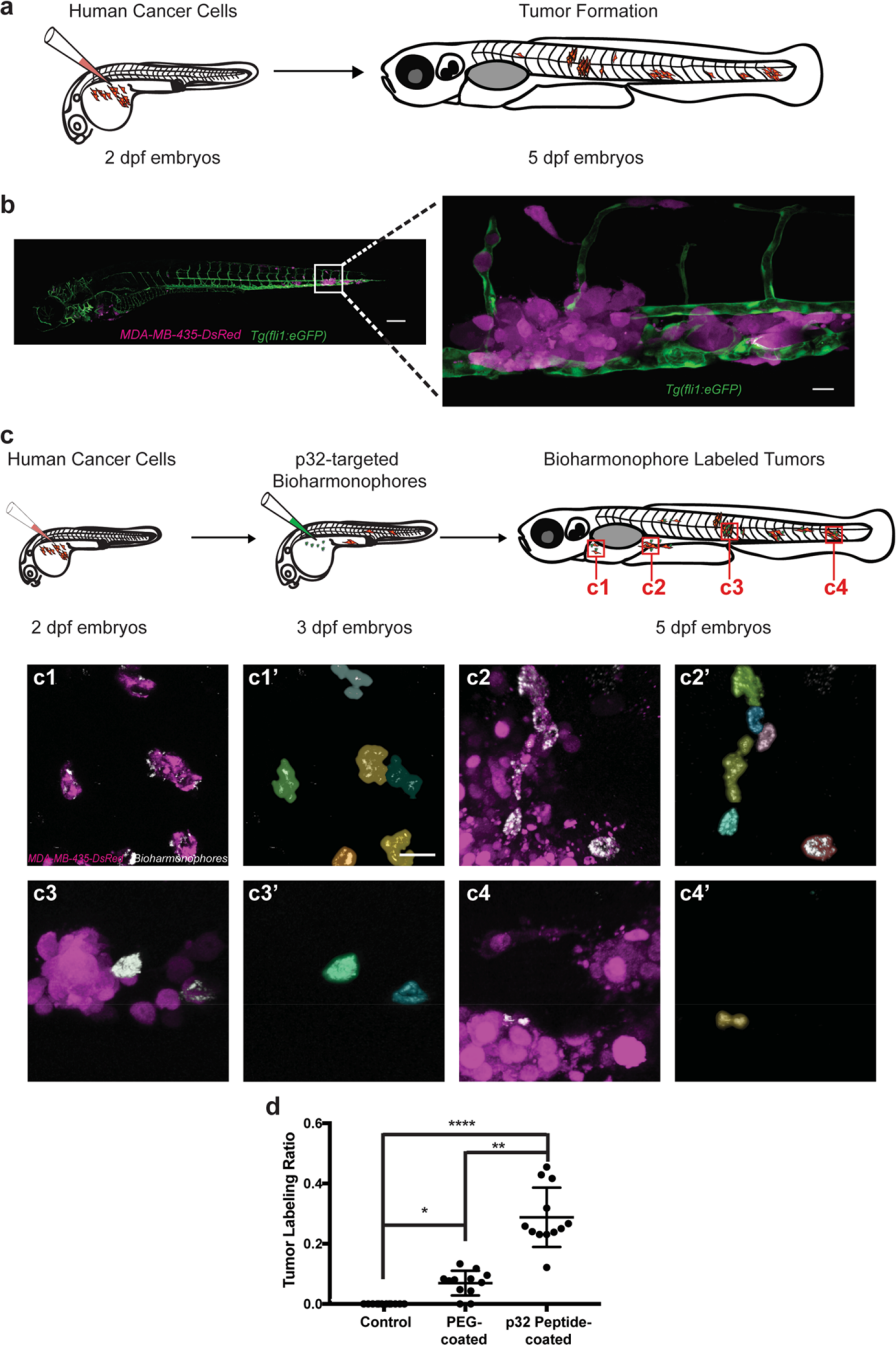
Bioharmonophores can be specifically targeted to single cancer cells *in vivo*. (a) Schematic showing the generation of a zebrafish cancer model by injecting MDA-MB-435-DsRed cancer cells into the Duct of Cuvier (DoC) at 2 dpf, resulting in tumors spread to multiple locations of the zebrafish body at 5 dpf. (b) Composite image of the cancer model (left) in a 5 dpf old zebrafish embryo. Close-up image of one of the tumor sites (right) reveals DsRed-labeled tumors (magenta), adjacent to the eGFP-labeled vasculature (green). (c) Schematic showing cancer cell injection of 2 dpf zebrafish embryos followed by bioharmonophore injection into DoC of 3 dpf zebrafish embryos and subsequent fluorescence and SHG imaging at 5 dpf. Red rectangles labeled as c1–4 denote the regions of interest that are illustrated in more detail. Individual panels showing the images of labeled cancer cells with the details of bioharmonophore (white) labeling down to single cancer cells (magenta) in solid tumors (c1–4). Colored cell boundary reconstruction of targeted cancer cells using the bioharmonophore SHG signal (c1′–4′). Note that cellular bioharmonophore distribution can in most cases predict cell morphologies. Scale bar, left panel 200 μm, right panel 20 μm (b); 15 μm (c). (d) Quantification of the fraction of SHG-labeled tumors as the ratio of labeled tumors to all tumors in a given zebrafish embryo after PEG- and p32 peptide-coated bioharmonophore injection, respectively. Each data point signifies one zebrafish. Note that active targeting with p32-coated bioharmonophores significantly increases the labeling efficiency (approximately 4-fold). Mean ± s.d. ****, *P* < 0.0001, **, *P* = 0.0063, *, *P* = 0.0470 (nonparametric Kruskal–Wallis test with Dunn’s post hoc multiple comparison). *N* = 12, pooled from 3 independent experiments.

To generate a highly aggressive cancer model that can be tracked over time, we injected a DsRed-expressing metastatic human melanoma cells (MDA-MB-435-DsRed) into the Duct of Cuvier (DoC) of zebrafish embryos at 2 dpf (days post fertilization) (Figure 4a).^29,30^ By 3 days after the injection, the resulting tumors spread to various locations in the body and were found next to blood vessels, which likely support the tumors with nutrients (Figure 4b).^31^

To demonstrate the specificity and efficiency of bioharmonophores as contrast agents that can accomplish resolution down to the single cell *in vivo*, we targeted bioharmonophores to tumor sites by taking advantage of the surface protein p32/gC1qR as a characteristic molecular marker for MDA-MB-435-DsRed cells.^32^ To this end, we functionalized bioharmonophores with a p32 targeting peptide, injected them into the DoC of zebrafish embryos at 3 dpf, 1 day after the embryos were injected with MDA-MB-435-DsRed cancer cells (Figure 4c), and assessed colocalization between cancer cells and bioharmonophore signal at 5 dpf (see Supplementary Note 2). In the absence of tumors, functionalized bioharmonophores did not cause clustering at the site of injection and were localized at different parts of injected zebrafish embryos (Supplementary Figure 17d–i), indicating good biodistribution. Without bioharmonophore injection, zebrafish as well as tumor sites did not produce any SHG background signal (Supplementary Figures 17a–c and 18a–c) with the exception of minimal endogenous SHG signal localized at the zebrafish tail,^33^ which was excluded from assessing specificity of tumor targeting (Supplementary Figure 19). In the case of passive targeting, zebrafish injected with PEG-coated bioharmonophores revealed limited tumor labeling (Supplementary Figure 18d–f), stemming from leaky blood vessels and enhanced permeability and retention effect (EPR).^34^ In contrast, we observed an increased accumulation of p32 peptide-targeted bioharmonophores within individual cancer cells at tumor sites throughout the zebrafish embryos (Figure 4c1–c4′), indicating that the tumor-labeling specificity and efficiency are highly dependent on the p32 targeting peptide. While p32 peptide-targeted bioharmonophores can extravasate to different tumor sites, not all the cancer cells were labeled (Figure 4c). This observation is potentially due to limited accessibility within densely packed solid tumors^35^ and the continued proliferation and metastasis of cancer cells between bioharmonophore administration and imaging (see Supplementary Note 2).

In order to determine the extent of labeling of targeted bioharmonophores in the xenograft zebrafish cancer model, we measured the colocalization of cancer cells with bioharmonophores at each tumor site for noninjected zebrafish as well as for zebrafish that were injected with p32 peptide-targeted and PEG-coated bioharmonophores, respectively (see Supplementary Note 2 and Figure 4c,d). The number of tumors were not significantly different between data sets (Supplementary Figure 20). The zebrafish cancer model injected with p32 peptide-targeted bioharmonophores had a significantly higher fraction of labeled tumors compared with noninjected and PEG-coated bioharmonophores (Figure 4d) due to our active targeting strategy. Overall, these results demonstrate that bioharmonophores exhibit high SNR and outstanding photostability for efficient labeling of individual cancer cells at multiple tumor sites *in vivo*.

## Conclusions

In summary, we introduced bioharmonophores as a class of imaging probes that retain all the photophysical advantages of previously introduced inorganic SHG nanoprobes. Because bioharmonophores consist of a biodegradable peptide core and a polymer shell, they can be metabolized within cells. Our experiments indicated a degradation time between 24 and 48 h after injection,^36−38^ which is similar to existing nanoparticle based and molecular imaging agents. Their relative stability before finding their target and degradation upon cellular entry could render bioharmonophores ideal contrast agent for clinical imaging applications. The straightforward implementation of robust functionalization strategies and a sufficiently high metabolic stability *in vivo* allowed us to target bioharmonophores with high detection sensitivity to individual tumor cells in live zebrafish embryos. With the recent development of nonlinear microendoscopes,^39,40^ bioharmonophores have the potential to emerge as superior contrast agents during image-guided surgery to help surgeons perform safer and highly precise tumor removal procedures. Moreover, their ability to target single cells could be exploited for detecting cancer stem cells, a subpopulation of cells responsible for tumorigenicity, invasion, and metastasis.^41^ Once successfully identified, the nonlinear signal of bioharmonophores could be used for light induced drug delivery or photodynamic therapy.^42^ By employing pulsed lasers in the infrared wavelength range that permit deep tissue penetration, targeted bioharmonophore signal could trigger highly localized cancer stem cell death.^43^ Finally, as the SHG signal intensity of bioharmonophore relies on the self-assembly behavior of each peptide,^44^ we anticipate that a screen for alternative peptide sequences may yield even brighter bioharmonophores that will potentially permit diagnosis with deep-tissue single-molecule detection sensitivity.

## Experimental Methods

### Formation of Large-Scale Peptide Nanotubes

Diphenylalanine (FF) and triphenylalanine (FFF) (Bachem) peptide assemblies were prepared as previously described.^45^ Briefly, peptides were freshly dissolved in hexafluoroisopropanol (Sigma) at 100 mg/mL concentration prior to experiments and diluted to 5 mg/mL final concentration in deionized water.

### Encapsulation of SHG-Active Peptide Assemblies

For the evaluation of different peptides and their SHG capabilities, 30 mg of PLLA was dissolved in 3 mL of chloroform (Sigma) along with 15 mg of triphenylalanine, 30 mg of pentalalanine (Bachem), and 30 mg of trileucine (Sigma) peptides in separate glass vials. The resulting suspension was mixed with aqueous SDS (Sigma) solution with a final 0.3% SDS concentration. A macroemulsion was obtained by stirring the samples at 1000 rpm for 1 h. Afterward, the samples were sonicated (Branson Sonifier) with a 1.5 in. tip at 70% power in a pulsed mode (30 s ON and 10 s OFF) for 2 min under ice cooling. The chloroform was evaporated from the obtained emulsions by stirring the samples at 500 rpm at 40 °C overnight. For the remaining experiments with triphenylalanine peptide containing bioharmonophores, the same protocol was followed unless stated otherwise. For probing the optimal conditions for nanoparticle formation, FFF peptide, PLLA, and SDS concentrations were varied as described in Supplementary Figures 6–8.

### Characterization of Encapsulated SHG-Active Peptide Assemblies

Produced samples were characterized using dynamic light scattering. Nanoparticle morphology, aggregation tendency, and the SHG signal intensity were evaluated using nonlinear microscopy. XRD patterns were obtained using a PANalytical X’PERT Pro powder diffractometer in Bragg–Brentano geometry and with Cu K-α1 radiation in grazing incidence geometry between 2–60 using a step size of 0.0167. The samples were air-dried on silicon single crystals, and four identical scans were obtained from each sample and summed up.

### SHG Polarimetry

The SHG polarimetry was performed on a wide-field SHG microscope (see the Supporting Information). A 1030 nm laser, pulse width 190 fs, and 200 kHz repetition rate (Pharos, Light Conversion), delivered 36 mW on the sample over a 150 μm fwhm diameter field-of-view (1 mJ·cm^–2^). Two non-colinear beams are incident on the sample, with a 30° opening angle. The SHG signal was detected in the phase matching direction (transmission). The image was recorded with an electron-multiplying intensified charge-coupled device (EM-ICCD) camera. Nonlinear polarimetry was performed by controlling and analyzing the polarization state of the illuminating and emitted beams. A polarization state generator, comprising a half- and a quarter-wave plate, was used. The polarization state of the emitted light was analyzed with a half-wave plate placed in the emission path, followed by a polarizing beam splitter.

### Second-Harmonic Spectroscopy Patterns

SHG emission pattern measurement was performed on a custom-build setup for this purpose (see the Supporting Information). Excitation was performed with a 1030 nm laser, pulse width 190 fs, and 200 kHz repetition rate, which delivered 60 mW on the sample, a cylindrical cuvette containing the solution, over a 36 μm focal spot (30 mJ·cm^–2^). The signal was detected with a rotating PMT and a filter (515 + 10, Chroma) at angles between −90 and 90. Both incident and detection polarizations was controlled.

### Stability of Biodegradable Bioharmonophores at Different pH Values

To evaluate how different pH values might influence the PLLA-coated peptide assemblies and their signal intensity, synthesized bioharmonophores were centrifuged for 3 min at 13500 rpm and resuspended in citric acid/Na_2_HPO_4_ buffer ranging from 4 to 7 pH values. The bioharmonophores were incubated for 72 h in the buffers containing 1% Tween 80 to prevent aggregation, and the signal intensity was monitored using nonlinear microscopy.

### Biodegradation of Bioharmonophores *in Vitro*

Bioharmonophores were centrifuged for 3 min at 13500 rpm and resuspended in 1% Tween 80 in 1X PBS. In order to assess proteinase K (Sigma) degradation, 1 mL of bioharmonophore suspension was incubated with 100 μg/mL final proteinase K concentration at 37 °C and the SHG signal intensity was measured every 2 h. Similarly, in order to assess how bioharmonophores were degraded using cellular content, an *ex vivo* biodegradation protocol was adapted based on the Rabbit Reticulocyte Lysate system (Promega). In a typical setup, 1 mL of bioharmonophore in 1% Tween 80 in 1X PBS was mixed with 25 mM phosphocreatine (Sigma), 10 μg/mL phosphocreatine kinase (Sigma), 1 mM ATP (Sigma), and 50 μL Rabbit Reticulocyte Lysate. The mixture was incubated at 37 °C, and the SHG signal intensity was monitored every 2 h.

### Biodegradable Bioharmonophore Functionalization

One milliliter of 1.5 mg/mL bioharmonophores was incubated with 1 mg of *Candida antarctica* Lipase B (Sigma) for 2 h, which hydrolyzes the PLLA polymer to increase the number of carboxyl groups. The bioharmonophore suspension was centrifuged at 13500 rpm for 3 min and resuspended in 1% Tween 80 in 1X PBS and mixed with 10 mg of *N*-(3-(dimethylamino)propyl)-*N*′-ethylcarbodiimide hydrochloride (EDC) (Sigma), 10 mg of *N*-hydroxysuccinimide (NHS) (Sigma), and 10 mg of methoxypolyethylene glycol amine 5000 Da (mPEG Amine) (Sigma) for 2 h. The suspension was centrifuged and resuspended in 1% Tween 80 in 1X PBS and stored at 4 °C prior to use.

For further functionalization experiments thiol-PEG-amine 2000 Da (SH-PEG-NH_2_) (Sigma) was used as a platform for bioorthogonal click chemistry. In a similar setup, 10 mg of EDC, 10 mg of NHS, and 10 mg of thiol-PEG-NH_2_ were incubated for 2 h. The suspension was centrifuged and resuspended in 1% Tween 80 in 1X PBS with methyltetrazine-PEG4-maleimide (Click Chemistry Tools) of 200 μM final concentration. The mixture was incubated for 2 h at room temperature, centrifuged, and resuspended in 1% Tween 80 in 1X PBS.

The other click chemistry pair *trans*-cyclooctene (TCO)-PEG3-maleimide (Click Chemistry Tools) (3 mM in 200 μL of PBS) was incubated for 2 h with cysteine-containing Tat or P32 targeting peptides (1 mM final concentration) depending on the application. The peptides were passed through Illustra Microspin G25 columns (GE Healthcare) to remove TCO-PEG3-maleimide.

A 200 μLportion of tetrazine-modified bioharmonophores was incubated with 20 μL of TCO-modified peptide for 2 h. The bioharmonophore suspension was washed with 1% Tween 80 in 1X PBS to remove unbound peptides and resuspended in 1X PBS to be immediately used for cell culture experiments.

### Cellular Degradation and Toxicity

MDA-MB-435-DsRed cancer cells were kindly gifted by Prof. R. Klemke. The cells were cultured at 37 °C, 5% CO_2_, in high glucose DMEM with GlutaMAX (10569010, Thermo Fisher), supplemented with 10% FBS (P40–37500, Pan Biotech) and 1X penicillin–streptomycin solution (15140122, Thermo Fisher). The cells were cultured on six-well plates (140675, Thermo Fisher) until they reached ∼80% confluency and were incubated with 400 μL Tat-derived cell penetrating peptide coated bioharmonophores overnight. The cells were washed with 1X PBS twice and detached using 0.05% Trypsin-EDTA (25300054, Thermo Fisher) in order to remove bioharmonophores that did not enter the cancer cells. Detached cells were centrifuged for 5 min at 500*g* to remove excess bioharmonophores that were not taken up, reseeded or ibiTreat-coated on eight-well slides (80826, Ibidi GmbH), and fixed after 6, 30, 54, and 78 h to monitor bioharmonophores degradation. The samples were then washed three times with 1X PBS and stained with the CellMask Orange Membrane dye (Invitrogen). The samples were washed again and imaged subsequently. To determine cell viability after treatment with functionalized bioharmonophores, trypan blue exclusion method was used. Briefly, cells in triplicates seeded in 96-well tissue culture plates (167008, Thermo Fisher) were exposed to varying concentrations of functionalized bioharmonophores for 48 or 72 h. After incubation, cells were washed with 1X PBS twice and detached as described above. Ten microliters of the cell suspension was then mixed with 10 μL of 0.4% Trypan Blue, and 4 μL of this mixture was added to the cell-counting slide (C10228, Thermo Fisher) and measured using Countess II Automated cell counter (Thermo Fisher). The viability was expressed as a fold difference of the untreated samples for each time point.

### Subcellular Localization of Bioharmonophores upon Cellular Uptake

HeLa cells were cultured at 37 °C, 5% CO_2_, in high glucose DMEM with GlutaMAX (10569010, Thermo Fisher), supplemented with 10% FBS (P40-37500, Pan Biotech) and 1× penicillin–streptomycin solution (15140122, Thermo Fisher). A 13 mm round coverslip was coated with 0.1% gelatin (G1393, Merck) for 1 h, and cells were seeded to reach around 70% confluency. The following day, 400 μL of freshly prepared thiol-PEG functionalized bioharmonophores^46,47^ (2 mg/mL) or an equal volume of 1X PBS (control) was added to the cells and incubated for 24 h. The cells were washed with 1X DPBS (containing Ca^2+^ and Mg^2+^, 14040133, Gibco) to remove the excess of not internalized bioharmonophores, fixed with 4% PFA for 15 min, permeabilized with 1X PBST (1X PBS containing 0.01% Tween20) for 15 min, blocked with 10% goat serum in 1X PBST for at least 1 h, and incubated overnight with mouse anti-LAMP2 (ab25631, Abcam, 1:200) in 5% goat serum at 4 °C. The next day, cells were rinsed with 1X PBST and incubated for 1 h with the goat antimouse Alexa488 conjugated secondary antibody (A28175, Thermo Scientific, 1:1000), washed thoroughly with 1X PBST, and mounted in mowiol mounting medium containing 2.5% v/v DABCO (290734, Merck).

### Toxicity Assay and Thioflavin T Staining

For toxicity assay, cells were grown in 96-well plates and were incubated with bioharmonophores at different concentrations for 48 and 72 h. After the incubation period, the cells were detached with trypsinization, and their viability was analyzed using Trypan Blue (Sigma) staining.

For thioflavin staining, cells were seeded in an eight-well chamber (ibidi) with 50% confluency. The cells were treated with either Amyloid Beta Peptide (Bachem) or 5 μL of bioharmonophores for 24 h and extensively washed with 1X PBS to remove excess peptides and bioharmonophores. To evaluate whether bioharmonophores induce fibril formation the cells were fixed with 4% PFA for 10 min and washed with 1X PBS three times. Afterward, 0.05% Thioflavin T (Sigma) solution was added to the sample for 8 min, and excess dye was washed with 80% ethanol for 5 min. The washing step was repeated three times, and the samples were imaged using confocal microscopy.

### Zebrafish Cancer Model and Bioharmonophore Targeting

Animal experiments and zebrafish husbandry were approved by the “Kantonales Veterinaeramt Basel-Stadt”. MDA-MB-435-DsRed cancer cells were injected into the Duct of Cuvier of *Tg(fli1:egfp)* zebrafish embryos at 2 days post fertilization (dpf). After injection, embryos were incubated for 1 h at 29 °C for recovery and cell transfer then verified by fluorescence microscopy. Fish harboring red cells were incubated at 35 °C essentially as described before.^29,30^ Fish were anaesthetized and embedded in low melting agarose as described previously^48^ and were imaged at 5 dpf for assessing cancer cell localization.

For targeting experiments, p32/gC1qR ligand-functionalized bioharmonophores were injected into the zebrafish embryos 24 h after cancer cell injection following the same procedure. *In vivo* bioharmonophores targeting was evaluated at 5 dpf using nonlinear laser scanning microscopy.

### Transmission Electron Microscopy

Bioharmonophore samples were spun down to remove aggregated nanoparticles at 3000 rpm for 3 min. Five microliters of the sample (*i.e.*, the supernatant of the centrifuged solution) was placed on a carbon coated grid (Quantifoil, D) previously glow-discharged for 30 s (Emitech K100X, GB). The drop was allowed to remain for 60 s; after this interval, excess fluid was drained along the periphery using a piece of filter paper followed by staining with 2% uranyl acetate for 1 and 15 s, respectively. Excess moisture was drained after each step, and when dry, the grid was examined in an FEI Morgagni 268 TEM operated at 100 kV.

### Nonlinear and Confocal Laser Scanning Microscopy

Bioharmonophores were immobilized in low melting agarose by mixing 200 μL of bioharmonophore with 100 μL of 1% SeaPlaque low melting agarose (Lonza) solution in eight-well imaging chambers (Lab-Tek). Imaging experiments were performed either on a Zeiss LSM 780 microscope (Carl Zeiss AG) equipped with a spectral GaAsP detector and a tunable two-photon laser source (Chameleon Ultra II, Coherent, Inc.) using an LD C-Apochromat 40×/1.1 water immersion objective lens (Carl Zeiss AG) or on a Leica SP5 microscope equipped with 2 Hybrid Detectors (HyD, Leica) and a tunable two-photon laser source (Mai-Tai 690-1020, Spectraphysics), using a HC PL Apochromat 63×/1.40 Oil immersion objective lens (Leica). Throughout the imaging experiments, bioharmonophores were illuminated with 850 nm incident wavelength, and the SHG signal was collected between 405 and 435 nm or with a GaAsP spectral wavelength detector for spectral measurements.

### Statistical Analysis

All numerical values represent mean ± s.d. Sample sizes (*n*) are given in the figure legends for each experiment. Each experiment was repeated at least three times. Normal distribution of data sets was established using the D’Agostino & Pearson omnibus normality test where *P* > 0.05 indicated Gaussian distribution. When all of the data sets had Gaussian distribution, one-way Anova was used for multiple comparisons followed by Tukey’s multiple comparisons. When one or more data sets showed a non-Gaussian distribution or high degree of variance as in the case of zebrafish tumor models, a Kruskal–Wallis test was applied along with Dunn’s multiple comparisons. For all statistical tests, a *P* value was reported, n.s., *P* > 0.05, *, *P* < 0.05, **, *P* < 0.005, ***, *P* < 0.001, ****, *P* < 0.0001. Second-order polynomial fit and one phase exponential decay values were calculated, and graphs were drawn using GraphPad Prism 6.

## Supporting Information Available

Supplementary figures as described in the text (Figures 1–20) and supplementary notes describing optical characterization of bioharmonophores and determination of the fraction of bioharmonophore-labeled tumors in a zebrafish cancer model (PDF)

## Notes

This publication was previously submitted to a preprint server: Sonay, A. Y.; Yaganoglu, S.; Konantz, M.; Teulon, C.; Sieber, S.; Jiang, S.; Behzadi, S.; Crespy, D.; Landfester, K.; Roke, S.; Langerke, C.; Pantazis, P. Biodegradable Harmonophores for Targeted High-Resolution *In Vivo* Tumor Imaging. *bioRxiv***2019**, 694760, https://www.biorxiv.org/content/10.1101/694760v1 (accessed 2021-02-02).
