# Supplementary material for: Biodegradable Harmonophores for Targeted High-Resolution In Vivo Tumor Imaging
Source: ACS Nano. 2021 Feb 25;15(3):4144–54. doi: 10.1021/acsnano.0c10634 (PMC8023799; doi:10.1021/acsnano.0c10634)
Supplement: Supplementary file 1 — nn0c10634_si_001.pdf [file nn0c10634_si_001.pdf]

Biodegradable Harmonophores for Targeted High-Resolution *In Vivo* Tumor Imaging

Ali Yasin Sonay[1,#], Konstantinos Kalyviotis[2], Sine Yaganoglu[1], Aysen Unsal[2], Martina Konantz[3], Claire Teulon[4], Ingo Lieberwirth[5], Sandro Sieber[6], Shuai Jiang[5], Shahed Behzadi[5], Daniel Crespy[5,7], Katharina Landfester[5], Sylvie Roke[4,8,9], Claudia Lengerke[3,10,&], Periklis Pantazis[1,2]*

[1]Department of Biosystems Science and Engineering (D-BSSE), Eidgenössische Technische Hochschule (ETH) Zurich, 4058 Basel, Switzerland

[2]Department of Bioengineering, Imperial College London, South Kensington Campus, London SW7 2AZ, UK

[3]Department of Biomedicine, University Hospital Basel and University of Basel, 4031 Basel, Switzerland

[4]Laboratory for Fundamental BioPhotonics, Institute of Bioengineering, School of Engineering, École Polytechnique Fédérale de Lausanne, 1015 Lausanne, Switzerland

[5]Max Planck Institute for Polymer Research, 55128 Mainz, Germany

[6]Division of Pharmaceutical Technology, Department of Pharmaceutical Sciences, University of Basel, 4031 Basel, Switzerland

[7]Department of Materials Science and Engineering, School of Molecular Science and Engineering, Vidyasirimedhi Institute of Science and Technology (VISTEC), Rayong 21210, Thailand

[8]Institute of Materials Science and Engineering, School of Engineering, École Polytechnique Fédérale de Lausanne, 1015 Lausanne, Switzerland

[9]Lausanne Centre for Ultrafast Science, École Polytechnique Fédérale de Lausanne, 1015 Lausanne, Switzerland

[10]Division of Hematology, University Hospital Basel, 4031 Basel, Switzerland

[#]Present address: Molecular Pharmacology Program, Memorial Sloan Kettering Cancer Center, 10065 New York, NY, USA

[&]Present address: Internal Medicine II, University Hospital Tübingen, 72076 Tübingen, Germany

[*]Email to: Periklis Pantazis, p.pantazis@imperial.ac.uk

This file includes:

Supplementary Figure 1	SHG signal generated by bioharmonophores with different self-assembling peptides.
Supplementary Figure 2	Synthesized bioharmonophores have similar structures when characterized using Transmission Electron Microscopy.
Supplementary Figure 3	X-ray diffraction analysis of different bioharmonophores have different diffraction patterns based on their unique peptide assembly.
Supplementary Figure 4	Inorganic SHG nanoprobes and biodegradable bioharmonophores show comparable signal intensities under the same illumination conditions.
Supplementary Figure 5	Triphenylalanine-based bioharmonophores generate bulk SHG response and the peptide assembly reveals a high internal order.
Supplementary Figure 6	Increasing peptide concentration for bioharmonophore production increases overall SHG signal of bioharmonophores yet affects their stability.
Supplementary Figure 7	Optimal surfactant concentration is essential to control bioharmonophore size and stability.
Supplementary Figure 8	Polymer concentration influences both the concentration of peptides inside bioharmonophores and the morphology of bioharmonophores.
Supplementary Figure 9	Proteinase K treatment induces degradation of bioharmonophores.
Supplementary Figure 10	Proteinase K induced bioharmonophore degradation characterized by Transmission Electron Microscopy at different time points.
Supplementary Figure 11	Bioharmonophores can be successfully coated with biomolecules using click chemistry.
Supplementary Figure 12	Lower pH does not affect the signal intensity of bioharmonophores.
Supplementary Figure 13	Bioharmonophores accumulate in lysosomes after cellular uptake and produce a bright SHG signal prior to biodegradation.
Supplementary Figure 14	Cell-free lysate treatment induces degradation of bioharmonophores.

Supplementary Figure 15	Bioharmonophores do not cause toxicity to cells and zebrafish embryos.
Supplementary Figure 16	Thioflavin T staining of DsRed expressing MDA-MB-435 cancer cells reveals that bioharmonophore incubation does not induce protein misfolding.
Supplementary Figure 17	Zebrafish embryos have minimal endogenous SHG signal and injected bioharmonophores do not aggregate *in Vivo.*
Supplementary Figure 18	Zebrafish cancer models without bioharmonophore injection and with PEG-coated-bioharmonophore injection do not show colocalization between DsRed-labeled cancer cells and SHG signal of bioharmonophores.
Supplementary Figure 19	Endogenous SHG signal present in the zebrafish tail was excluded from assessing bioharmonophore targeting efficiency.
Supplementary Figure 20	The number of tumors does not significantly differ between zebrafish cancer model groups used for bioharmonophore labeling experiments.
Supplementary Note 1	Optical characterization of bioharmonophores.
Supplementary Note 2	Determining the fraction of bioharmonophore-labeled tumors in a zebrafish cancer model.
Supplementary References	

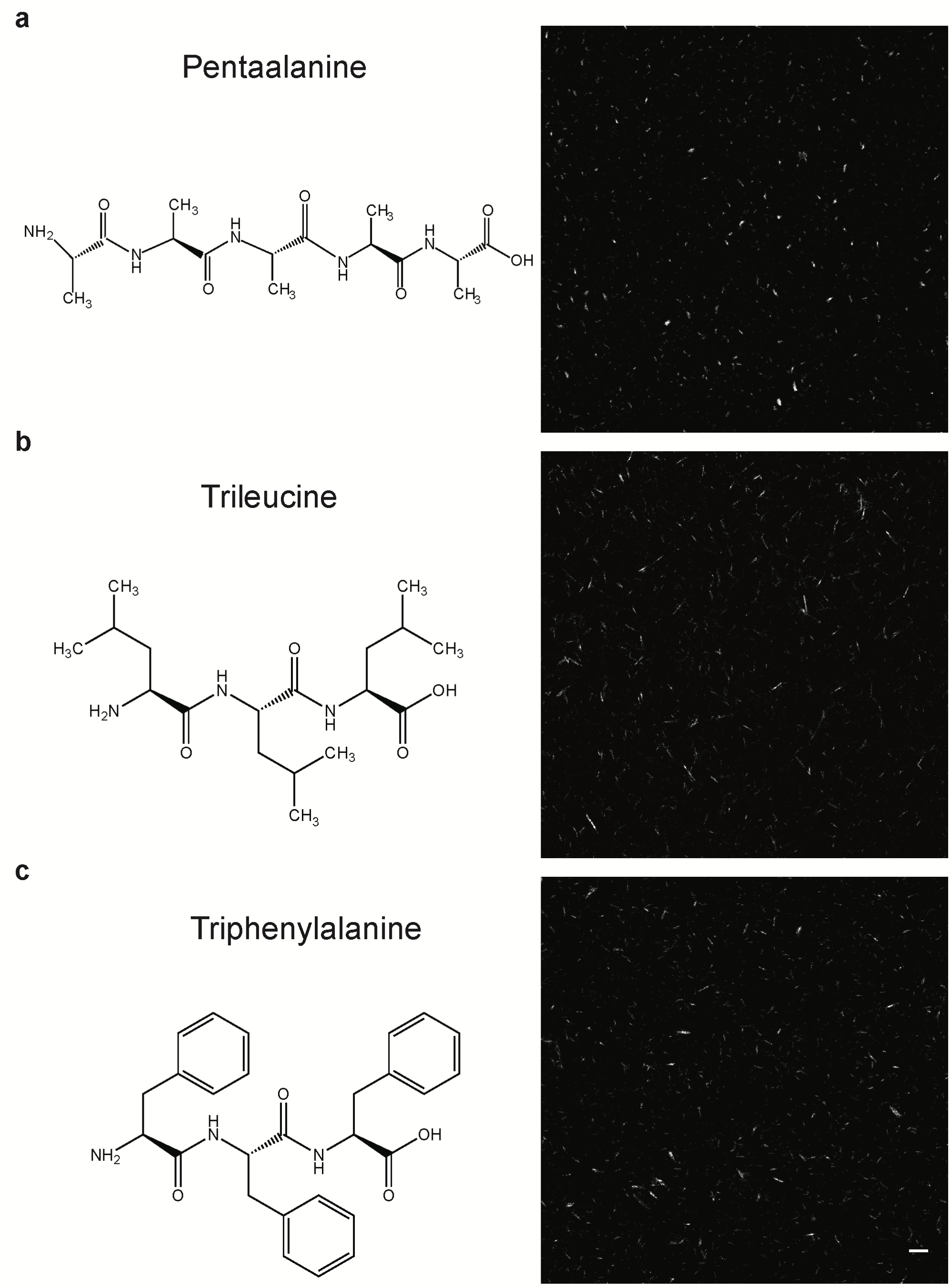

Supplementary Figure 1. SHG signal generated by bioharmonophores with different self-assembling peptides. Chemical structure of a, Pentaalanine, b, Trileucine, and c, Triphenylalanine peptides (left) and corresponding images capturing their SHG signal after encapsulation (right). Scalebar, 10 µm.

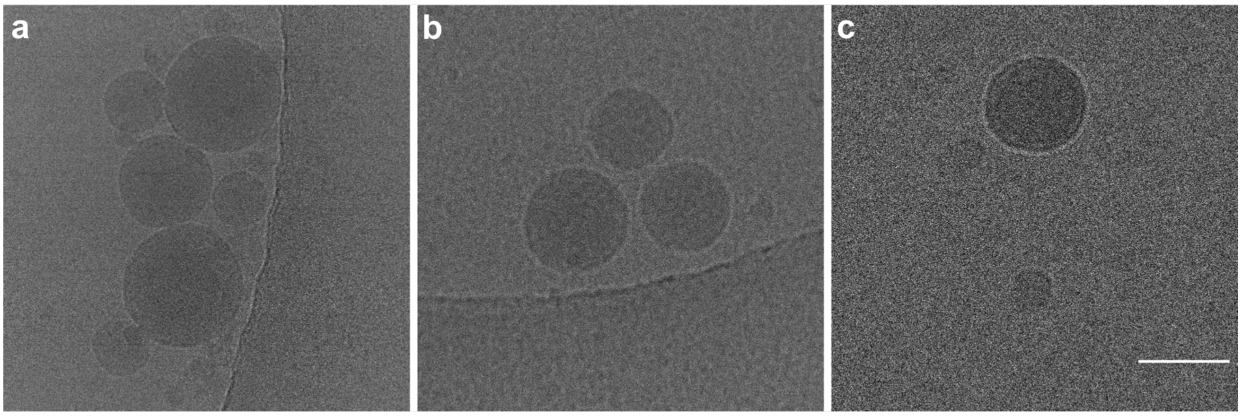

Supplementary Figure 2. Synthesized bioharmonophores have similar structures when characterized using Transmission Electron Microscopy. Representative images of bioharmonophores with different peptide cores: a, Triphenylalanine, b, Trileucine, and c, Pentaalanine. Scalebar, 100 nm.

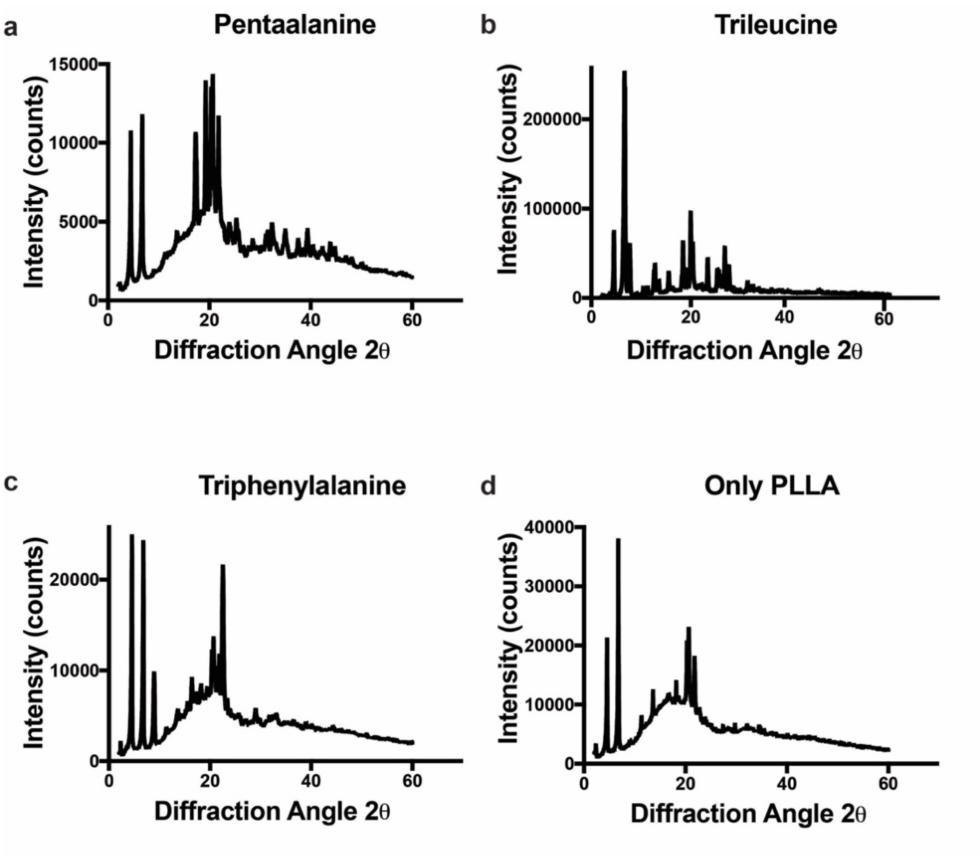

Supplementary Figure 3. X-ray diffraction analysis of different bioharmonophores have different diffraction patterns based on their unique peptide assembly. XRD patterns of bioharmonophores with different peptide cores show specific diffraction patterns and high degree of internal order for peptide assemblies a, Pentaalanine, b, Trileucine, c, Triphenylalanine. d, Bioharmonophores without a peptide core display significant background with overlapping peaks, interfering with a definitive crystallographic analysis of peptide core.

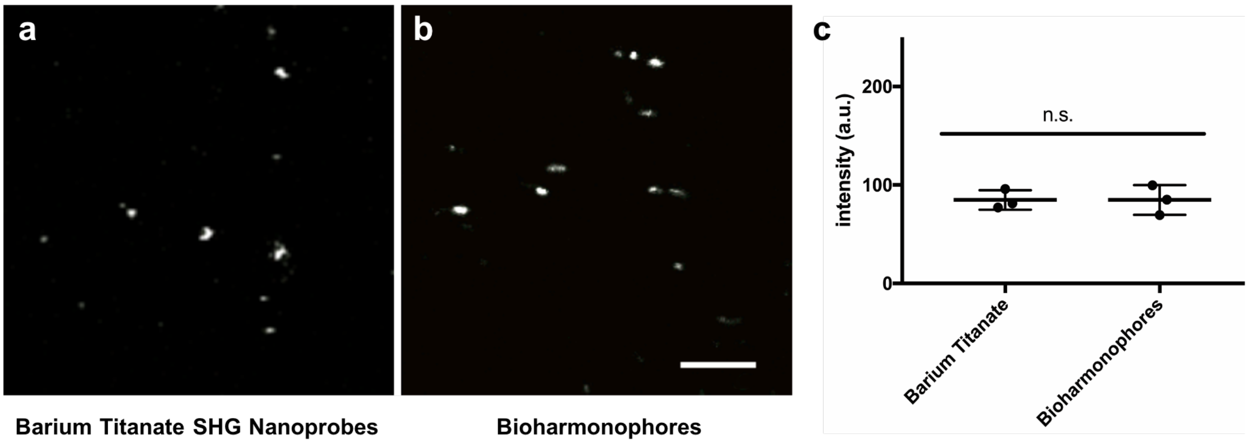

Supplementary Figure 4. Inorganic SHG nanoprobes and biodegradable bioharmonophores show comparable signal intensities under the same illumination conditions. Similar size ranges of barium titanate SHG nanoprobes and bioharmonophores were illuminated with the same laser intensity and their SHG signal was monitored using the same detection settings. a,b, SHG images of both nanoparticles display comparable particle brightness for inorganic SHG nanoprobes (left) and biodegradable bioharmonophores (right) regarding signal-to-noise ratio and single particle sensitivity. Scalebar, 10 µm. c, Quantification of single particle intensities exhibit no statistically significant difference between the signal intensities of SHG nanoprobes and bioharmonophores, respectively.

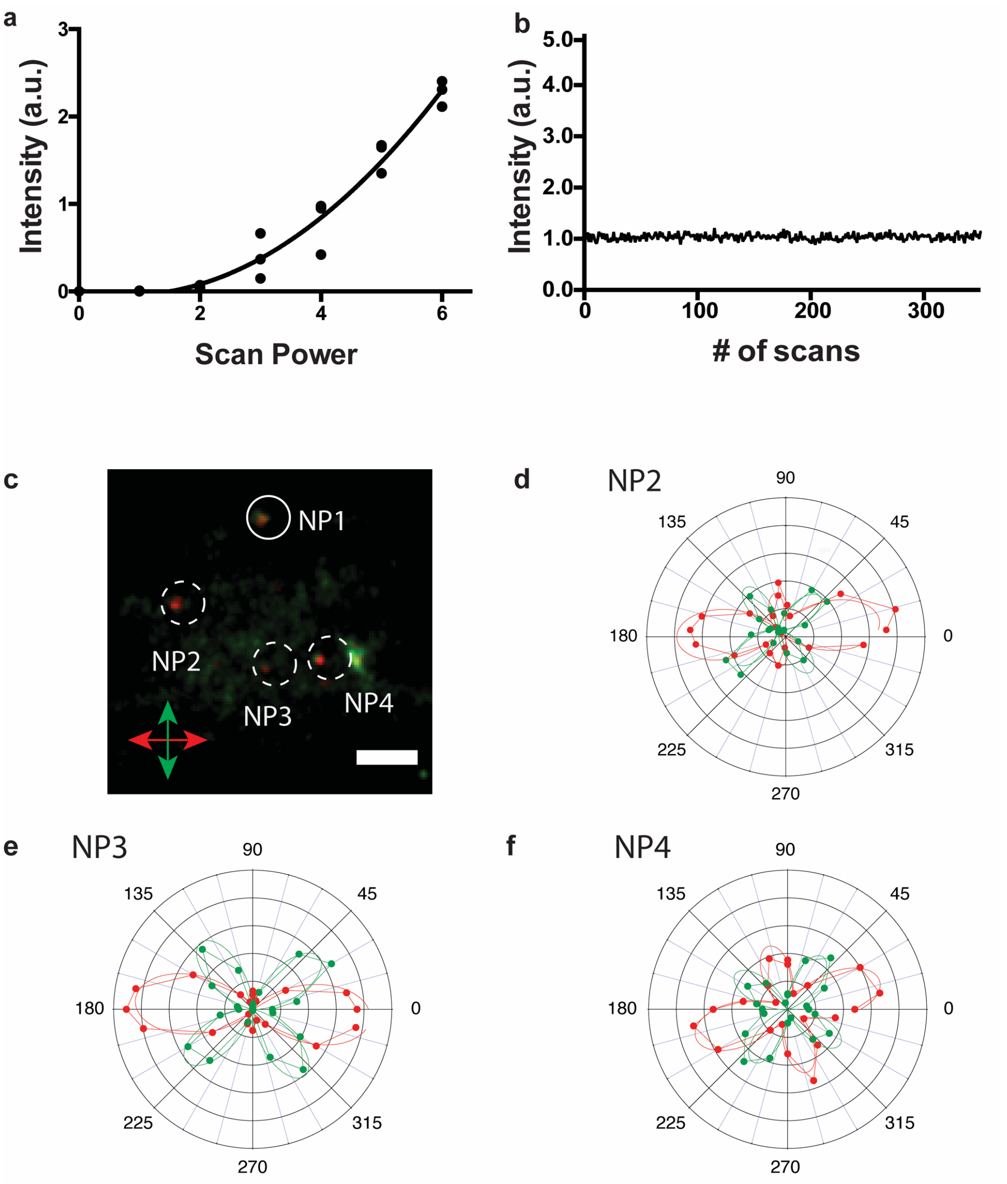

Supplementary Figure 5. Triphenylalanine-based bioharmonophores generate bulk SHG response and the peptide assembly reveals a high internal order. a, Increasing incident laser power leads to a quadratic increase of the SHG signal intensity of bioharmonophores (n=3). Mean values were fitted using a second order polynomial. b, Bioharmonophores do not display blinking when they are illuminated for an extended period of time with low-intensity levels of 850 nm light and a scanning speed of 5 frames per second. c, Bioharmonophores probed with SHG polarimetry are shown in white circles. A stack of images with different incident polarizations is taken with a given detected polarization (either X in red or Y in green). Here, the sum of images for all incident polarizations for detection along X (red) or Y (green) is depicted. Scalebar, 10 µm. d, e, f, SHG intensity *vs*. incident polarization angle displayed for the 4 highlighted bioharmonophores denoted as NP for nanoparticle. The curve for NP1, highlighted by the solid white circle, is displayed in Figure 2c. The experimental curves are dotted lines, the corresponding fitted curves, assuming monoclinic (C_2) symmetry, are solid lines.

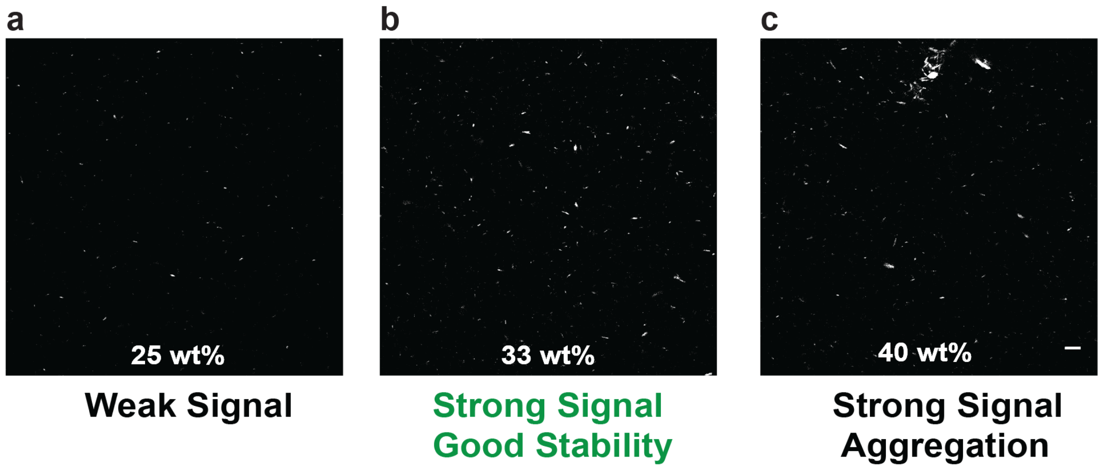

Supplementary Figure 6. Increasing peptide concentration for bioharmonophore production increases overall SHG signal of bioharmonophores yet affects their stability. Representative images illustrating the morphology, aggregation behavior, and signal intensity of bioharmonophores synthesized using different triphenylalanine peptide concentrations with a, 25 wt% peptide generating little signal due to low amount of peptide, b, 33 wt% peptide showing the optimal synthesis method based on size and stability, and c, 40 wt% peptide showing large aggregates due to aggregation behavior of the peptide. Scalebar, 10 µm.

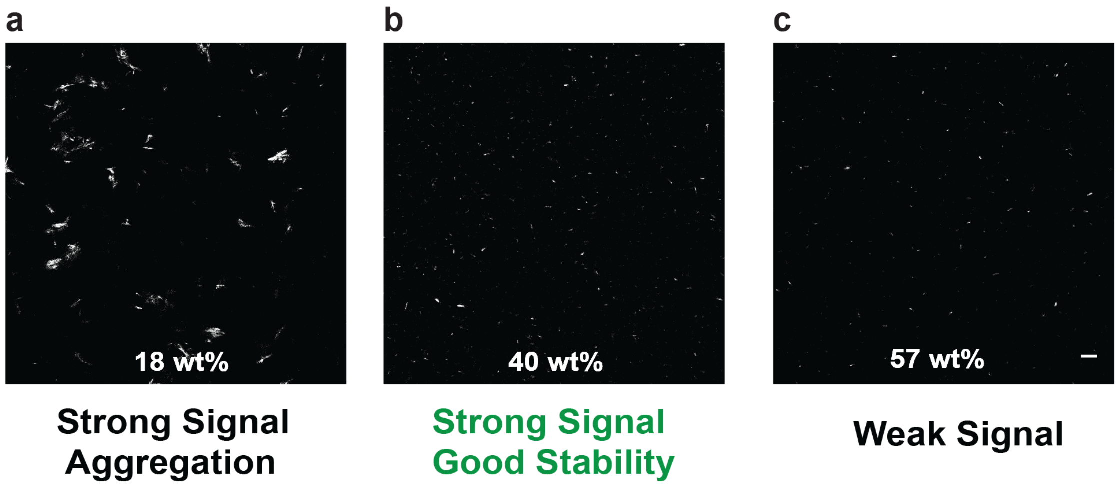

Supplementary Figure 7. Optimal surfactant concentration is essential to control bioharmonophore size and stability. Representative images illustrating the morphology, aggregation behavior, and signal intensity of bioharmonophores synthesized using different concentrations of SDS surfactant with a, 18wt% surfactant concentration leading to large aggregates, b, 40 wt% surfactant concentration as the optimal synthesis method based on size and stability, and c, 57 wt% surfactant concentration showing small nanoparticles with very little signal. Scalebar, 10 µm.

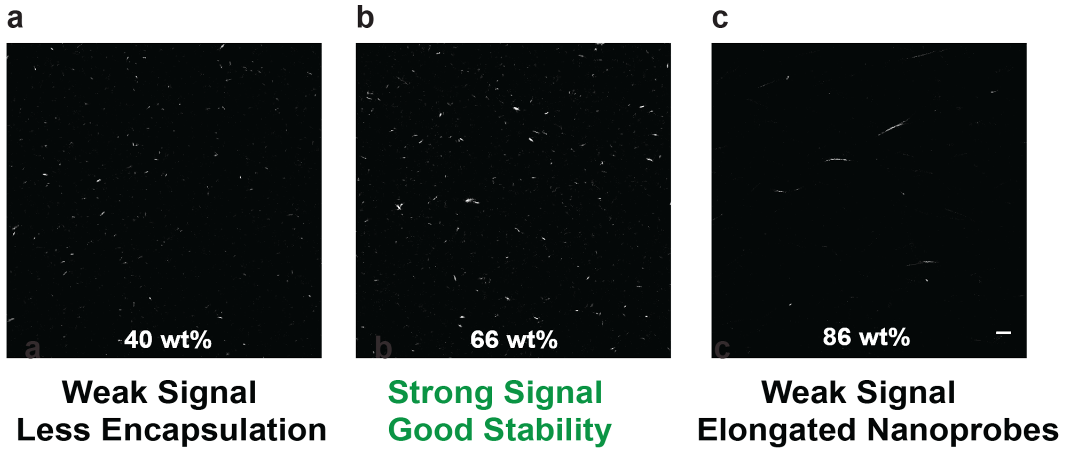

Supplementary Figure 8. Polymer concentration influences both the concentration of peptides inside the bioharmonophores and the morphology of bioharmonophores. Representative images illustrating the morphology, aggregation behavior and signal intensity of bioharmonophores synthesized using different Poly(*L*-Lactic acid) (PLLA) concentrations with a, 40 wt% PLLA concentration leading to limited amount of encapsulated peptides within the polymers, b, 66 wt% PLLA concentration as the optimal synthesis method based on signal intensity and bioharmonophore morphology, and c, 86 wt% PLLA concentration yielding elongated nanoparticles of diminished signal due to a smaller number of peptides contained within each bioharmonophore. Scalebar, 10 µm.

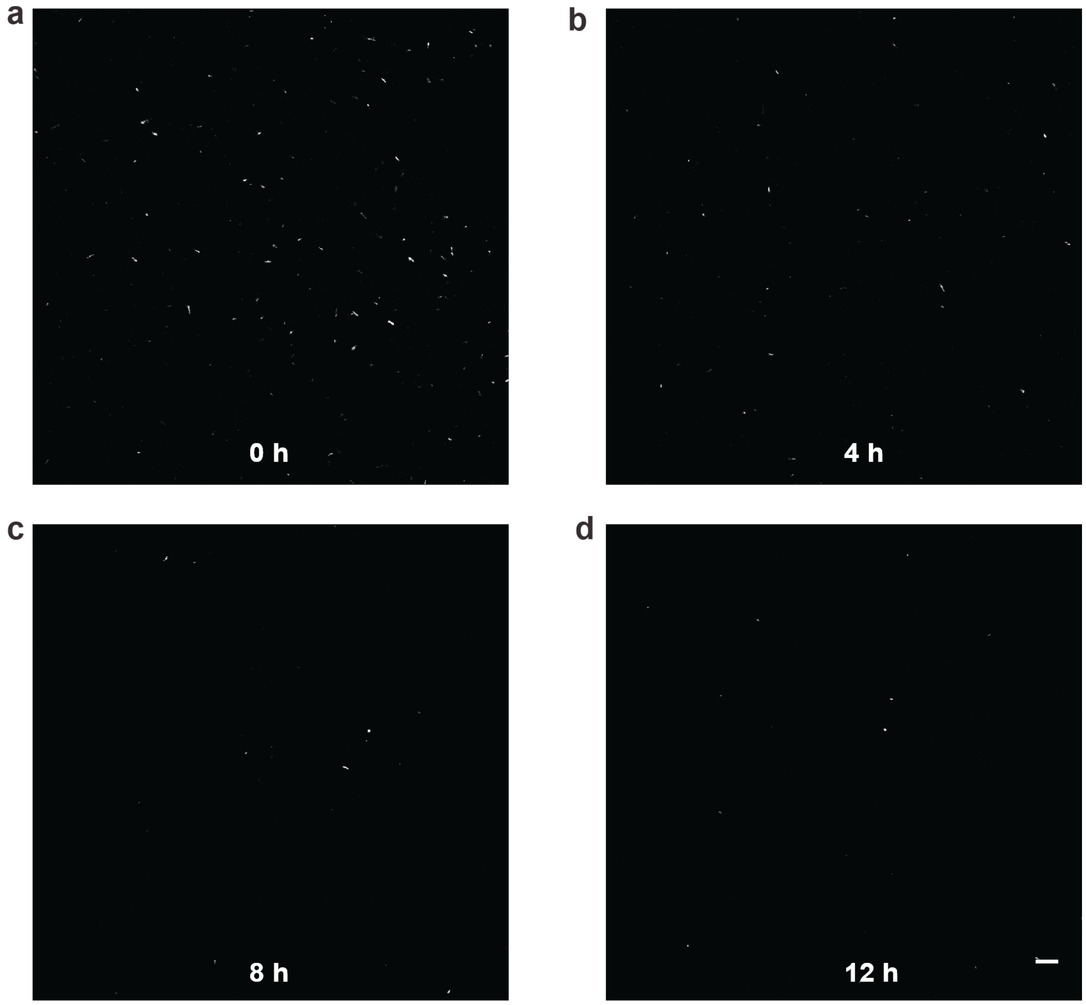

Supplementary Figure 9. Proteinase K treatment induces degradation of bioharmonophores. Representative images of Proteinase K treated bioharmonophores and their degradation monitored by changes of their SHG signal over time at a, 0 hours, b, 4 hours, c, 8 hours, and d, 12 hours. Scalebar, 10 µm.

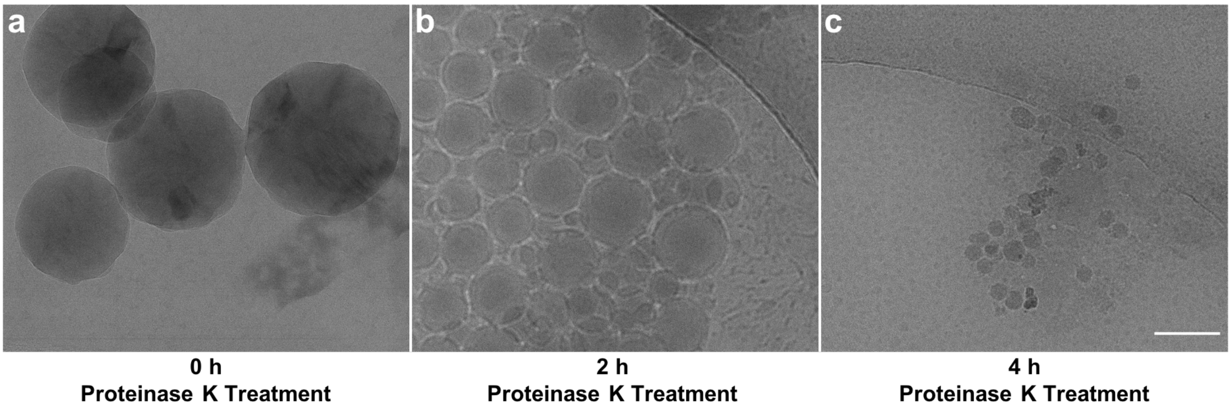

Supplementary Figure 10. Proteinase K induced bioharmonophore degradation characterized by Transmission Electron Microscopy at different time points. Representative images of bioharmonophores treated with Proteinase K and imaged at different time points. a, 0 hours, b, 2 hours, and c, 4 hours of Proteinase K treatment. The magnification is the same for the different timepoints, illustrating the degradation of bioharmonophore structures into smaller fragments within 4 hours. Scalebar, 100 nm.

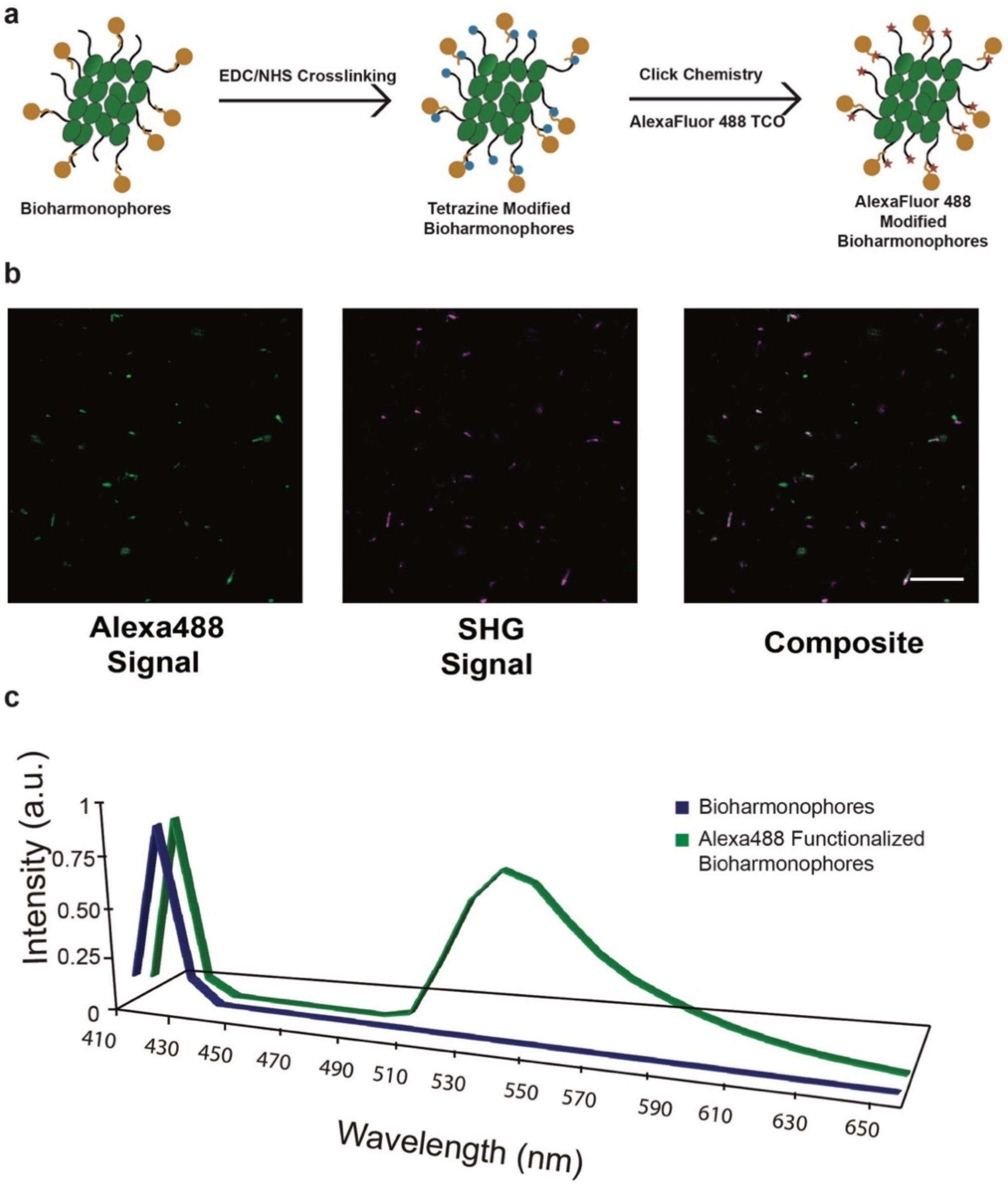

Supplementary Figure 11. Bioharmonophores can be successfully coated with biomolecules using click chemistry. a, Scheme showing the functionalization procedure to introduce tetrazine (Tz) groups on the bioharmonophore surface and its subsequent linkage with AlexaFluor488-trans-cyclooctene (TCO). The TCO-tetrazine pair undergoes a click reaction within 1 hour at room temperature with high specificity. b, AlexaFluor 488 signal (red) immobilized on the surface of bioharmonophores (left), SHG signal of bioharmonophores (center); and composite image showing a high degree of colocalization between Alexa488 and SHG signal (right). c, Spectral scan comparing the Alexa488-treated bioharmonophore with the untreated sample. Alexa488-functionalized samples retain the characteristic Alexa488 signal profile next to the SHG signal profile. Scalebar, 5 µm.

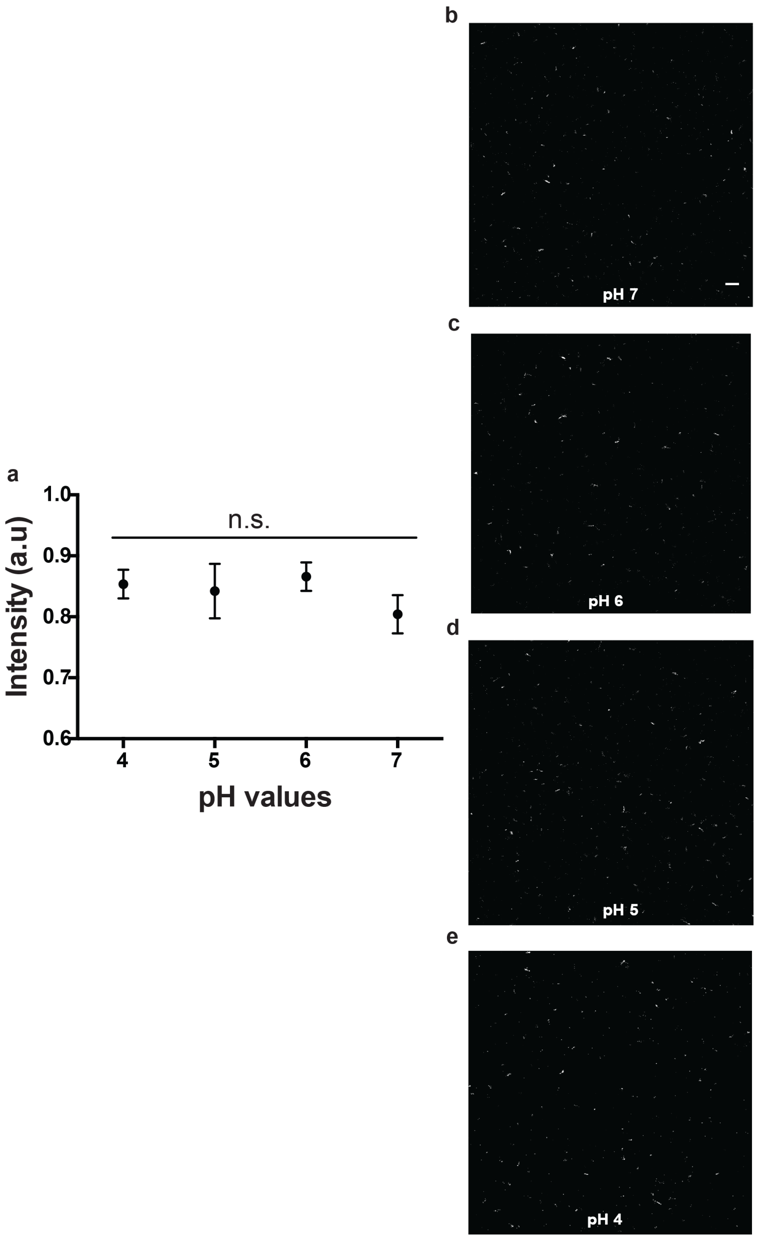

Supplementary Figure 12. Lower pH does not affect the signal intensity of bioharmonophores. a, Effect of different pH values on the SHG signal intensity when bioharmonophores were incubated at different pH values for 72 hours (n=5). Representative images showing SHG signal intensity after bioharmonophores were incubated in buffers at b**,** pH 7, c, pH 6, d, pH 5, and e, pH 4. Scalebar, 10 µm. Mean ± s.d. n.s., not significant (Ordinary one-way ANOVA with Tukey's multiple comparisons).

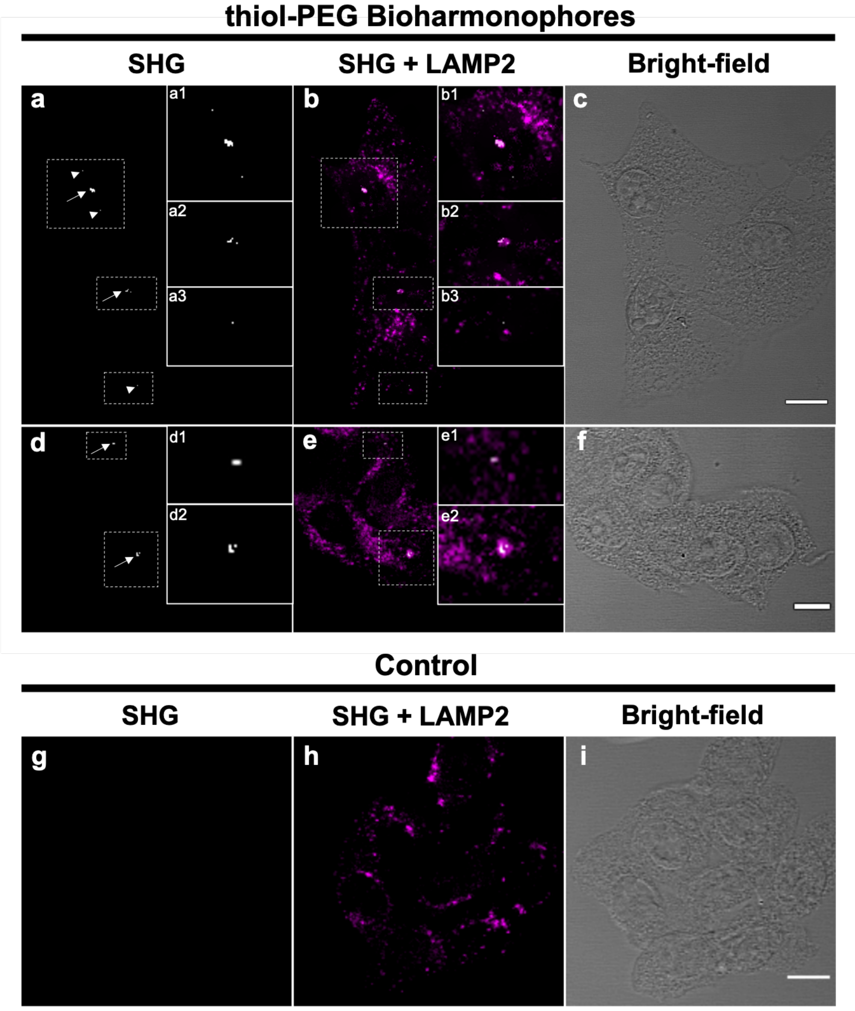

Supplementary Figure 13. Bioharmonophores accumulate in lysosomes after cellular uptake and produce a bright SHG signal prior to biodegradation. Representative images of bioharmonophores and lysosomes localisation in HeLa cells incubated with thiol-PEG functionalized bioharmonophores (a-f) or 1X PBS (g-i) for 24 hours followed by immunofluorescence staining for LAMP2. (a, d) SHG signal by internalized bioharmonophores. (b, e) Overlay images of SHG signal and fluorescent LAMP2 immunostaining of late endosomes/lysosomes that demonstrate bioharmonophores accumulation in LAMP2 positive vesicles. (c, f) Brightfield images of cells in a-b and d-e. (g, h) No SHG signal is detected in the 1X PBS control. (i) Brightfield image of the cells in g-h. Scalebars, 10µm.

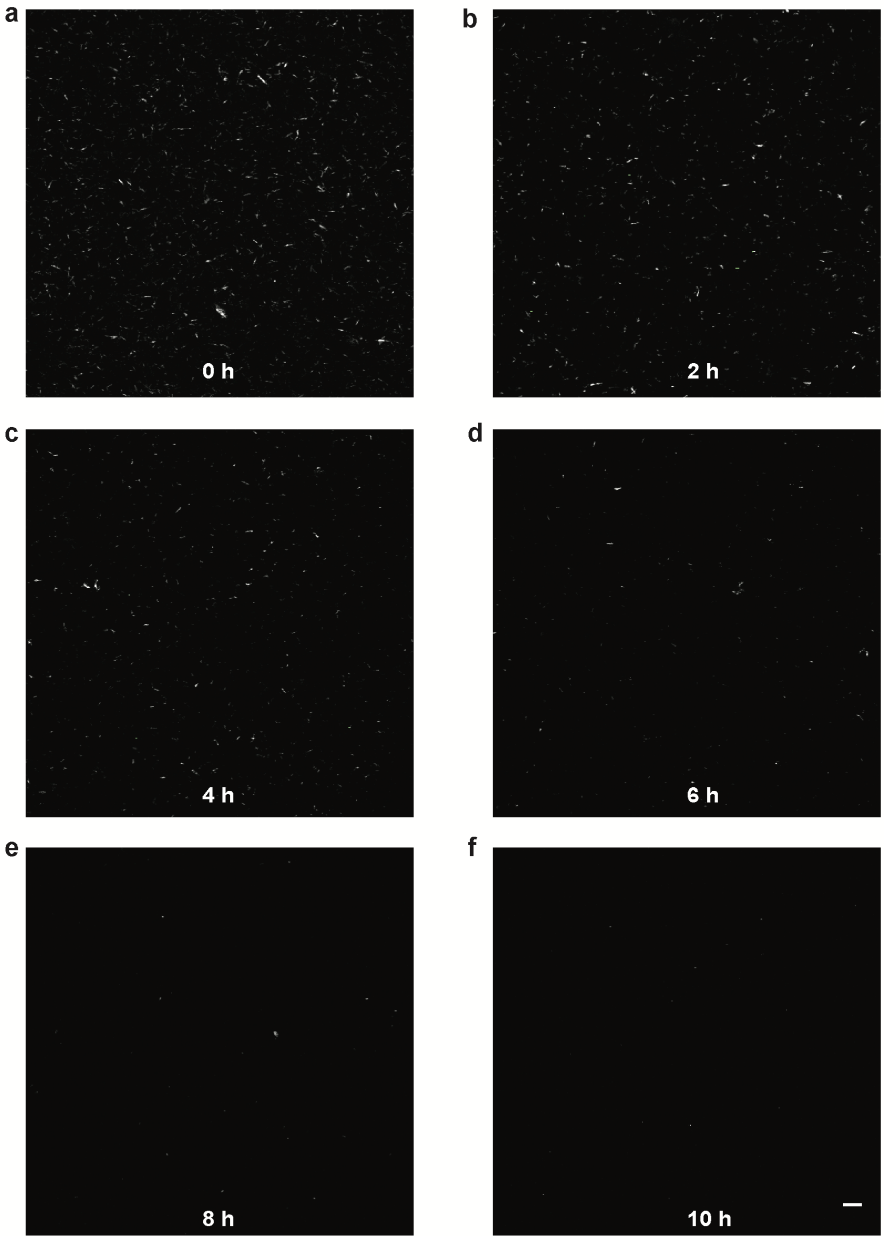

Supplementary Figure 14. Cell-free lysate treatment induces degradation of bioharmonophores. Representative images of bioharmonophores and their degradation using a cell-free reticulocyte lysate-based degradation protocol. Degradation is monitored by changes in the SHG signal over time at a, 0 hours, b**,** 2 hours, c, 4 hours, d, 6 hours, e, 8 hours, and f, 10 hours. Scalebar, 10 µm.

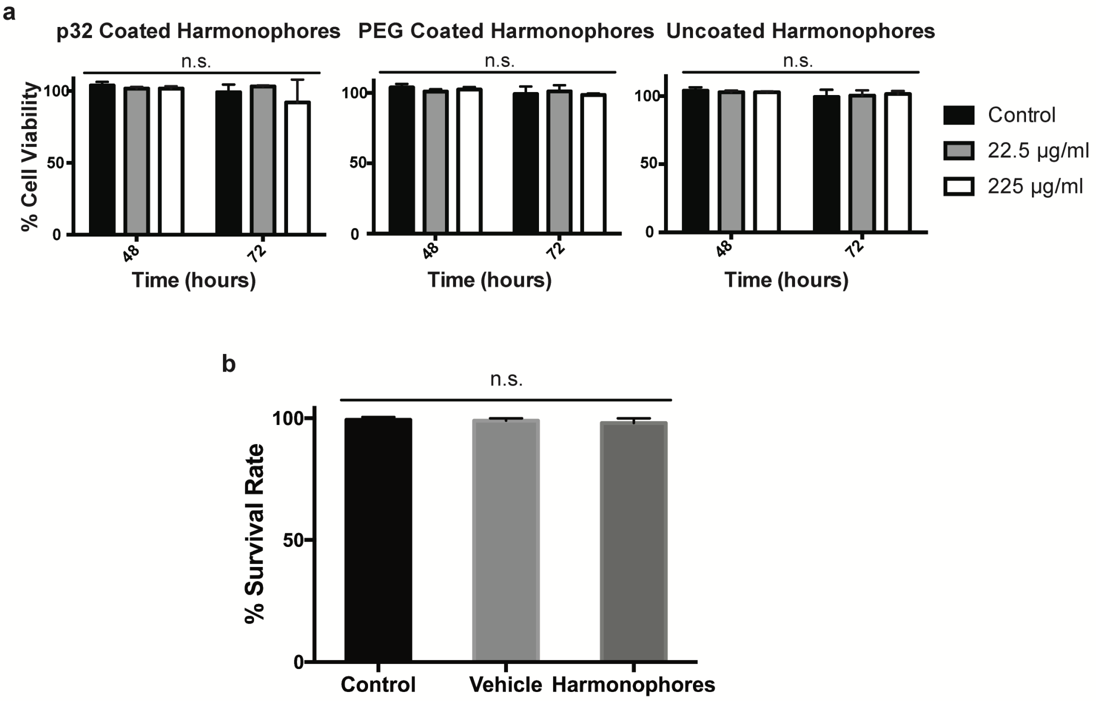

Supplementary Figure 15. Bioharmonophores do not cause toxicity to cells and zebrafish embryos. a, From left to right, bioharmonophores were coated with p32 peptide, PEG, and PLLA and incubated with cells for 48 and 72 hours. Under these conditions, they do not affect cell viability (n=3). b**,** Bioharmonophores were injected into the zebrafish blood stream at 2 dpf and their survival rate was recorded at 5 dpf. Under these conditions, they do not harm the zebrafish embryos (N=75, pooled from 3 independent experiments). Mean ± s.d., n.s., not significant (Ordinary one-way ANOVA with Tukey's multiple comparisons).

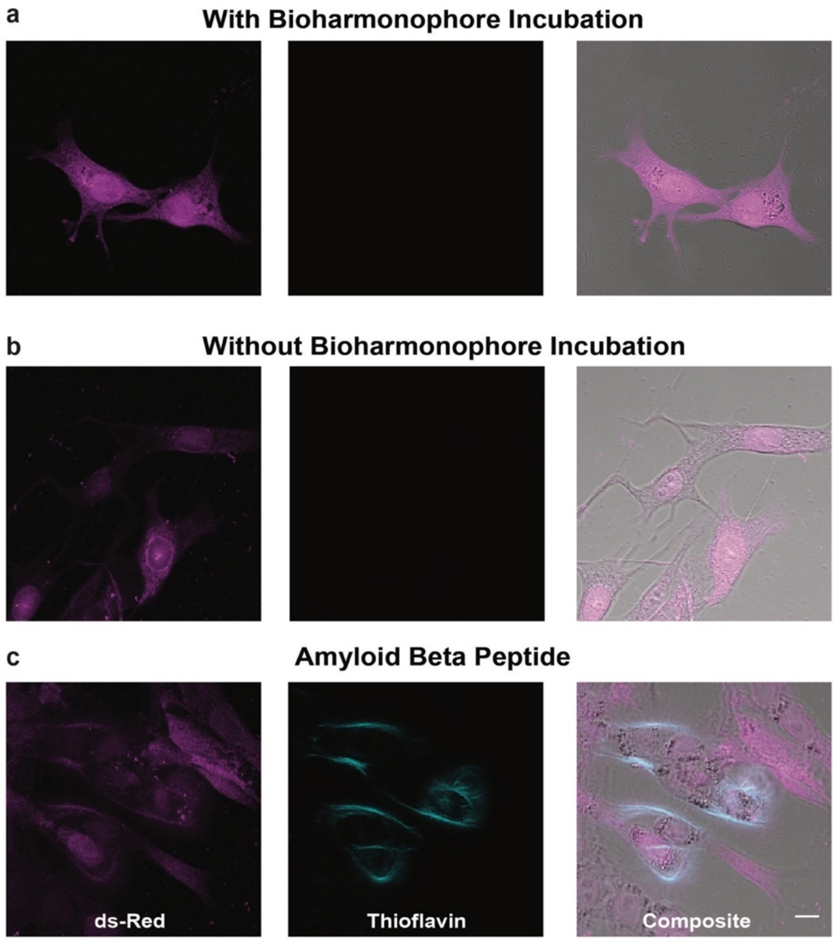

Supplementary Figure 16. Thioflavin T staining of DsRed expressing MDA-MB-435 cancer cells reveals that bioharmonophore incubation does not induce protein misfolding. a, DsRed-expressing cancer cells were incubated with bioharmonophores, cultured for 1 day, and stained with Thioflavin T to detect any bioharmonophore-induced aggregation toxicity. (left) DsRed, (center) Thioflavin T signal, (right) composite image. b, DsRed-expressing cancer cells cultured without bioharmonophores incubation, followed by Thioflavin T staining. (left) DsRed, (center) Thioflavin T signal, (right) composite image. The absence of Thioflavin T signal indicates that cells incubated with bioharmonophores do not display any protein aggregation-induced toxicity, similar to control cells in a. c, As a positive control, cells were incubated with amyloid beta protein for 24 hours and stained following the same protocol. Scalebar, 10 µm.

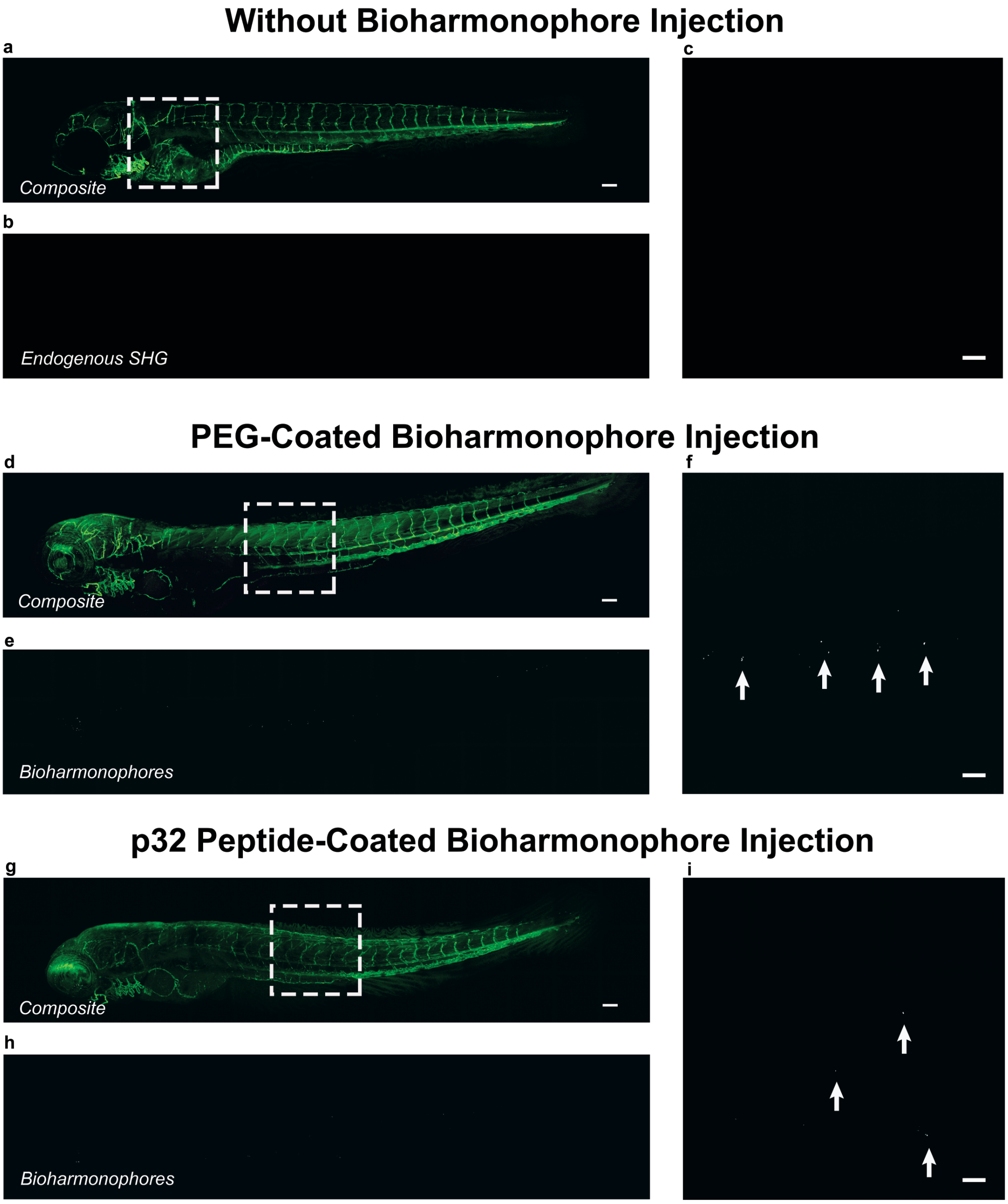

Supplementary Figure 17. Zebrafish embryos have minimal endogenous SHG signal and injected bioharmonophores do not aggregate *in Vivo*. a, Composite image of a *Tg(fli1:egfp)* zebrafish embryo (5 dpf) with blood vessels in green and endogenous SHG signal in white. b, Endogenous SHG signal originating from the zebrafish embryo is minimal. c, Enlarged view of tissue region enclosed in a, demonstrating no presence of endogenous SHG signal. d,e, Zebrafish embryo injected with PEG-coated bioharmonophores at 3 dpf and imaged for fluorescence and SHG signal at 5 dpf shows no aggregation of bioharmonophores. f, Enlarged view of tissue region enclosed in d reveals presence of bioharmonophores (arrows) in the zebrafish. g,h, Zebrafish embryo injected with p32 targeting peptide-coated bioharmonophores at 3 dpf and imaged for fluorescence and SHG signal at 5 dpf displays no bioharmonophore aggregation. i, Enlarged view of tissue region enclosed in g reveals presence of bioharmonophores (arrows) in the zebrafish. Representative image from n=12 pooled from 3 independent experiments. a,d,g, Scalebar, 100 µm. c, f, i, Scalebar, 50 µm.

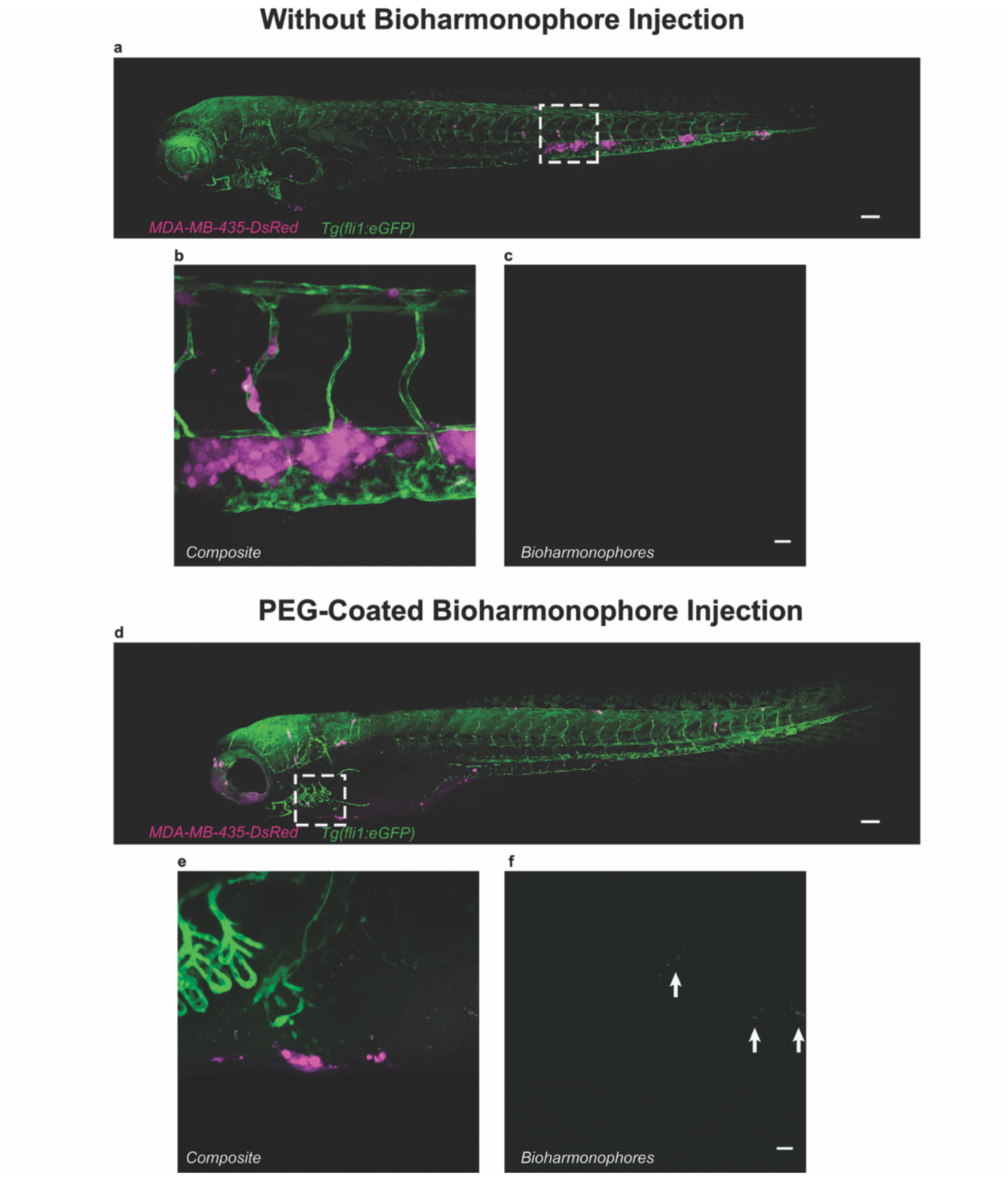

Supplementary Figure 18. Zebrafish cancer models without bioharmonophore injection and with PEG-coated-bioharmonophore injection do not show colocalization between DsRed-labeled cancer cells and SHG signal of bioharmonophores. *Tg(fli1:egfp)* zebrafish embryos were injected with Ds-Red expressing cancer cells at 2 dpf and imaged at 5 dpf with and without bioharmonophore injection, respectively. a, Whole zebrafish embryo showing the localization of tumors. b, Enlarged view of tissue region enclosed in a, composite image showing individual tumors. c, SHG channel reveals lack of bioharmonophore signal in the same enlarged region. d, Zebrafish cancer model injected with cancer cells at 2 dpf and with PEG-coated bioharmonophores at 3 dpf showing the localization of different tumors. e, Enlarged view of tissue region enclosed in d, composite image showing individual tumors and bioharmonophores. f, SHG signal of bioharmonophores does not colocalize with DsRed signal. Representative image from n=12 pooled from 3 independent experiments. a, d, Scalebar, 100 µm. c, f, Scalebar, 20 µm.

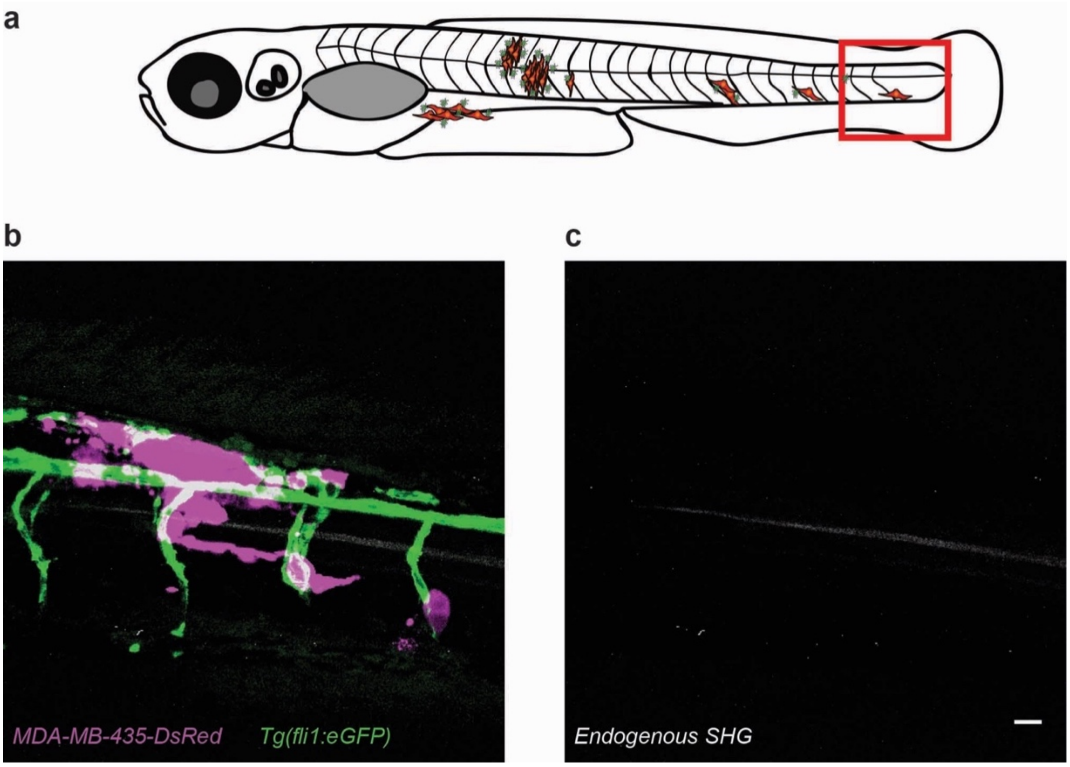

Supplementary Figure 19. Endogenous SHG signal present in the zebrafish tail was excluded from assessing bioharmonophore targeting efficiency. a, Scheme of a zebrafish embryo displaying the localization of endogenous SHG signal highlighted by a red square. b, Composite figure displaying green fluorescent signal of the vessels of *Tg(fli1:egfp)* zebrafish embryos and DsRed expressing cancer cells. c, SHG signal from endogenous structures possibly from collagen assemblies during bone formation, Scalebar, 20 µm.

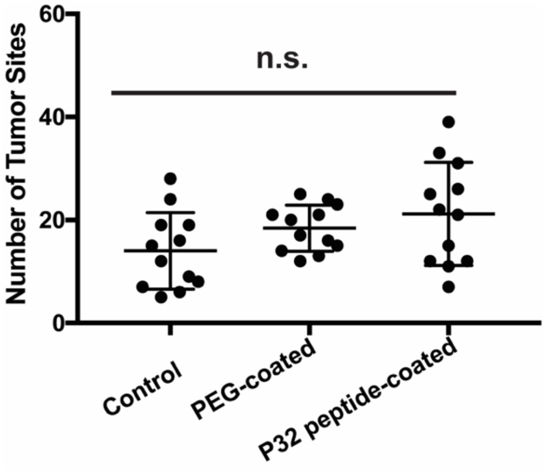

Supplementary Figure 20. The number of tumors does not significantly differ between zebrafish cancer model groups used for bioharmonophore labeling experiments. Number of tumors were quantified in individual zebrafish embryos that were injected with PEG- or p32 targeting peptide-coated bioharmonophores. While there is heterogeneity in the number of tumor sites, no statistically significant difference was found between the populations that were used for quantifying the extent of bioharmonophore labeling. Mean ± s.d. not significant (n.s.) $P > 0.05$ (non-parametric Kruskal-Wallis test with Dunn's post hoc multiple comparison). Each dot represents a total number of tumors from whole zebrafish embryo, N=12, pooled from 3 independent experiments.

Supplementary Note 1 – Optical characterization of bioharmonophores.

Descriptions of Optical Setups

SHG Polarimetry

Polarimetry was performed on a custom-built wide-field SHG microscope.[1] The light source was an amplified laser system (Pharos SP-1.5, LightConversion) delivering 190 fs laser pulses centered around 1036 nm (±6 nm) with a beam diameter of 3.7 mm and a maximum output power of 6 W, at variable repetition rates between 1 kHz-1 MHz. The laser system was operated at 200 kHz, and delivered 36 mW on the sample (1 mJ/cm^2). The field-of-view had a FWHM diameter of 150 µm. All microscope mirrors were protected silver mirrors (Thorlabs, PF10-03-P01). The (achromatic) lenses and other optical elements on the illumination path were near-infrared antireflection coated (Thorlabs, B), whereas the optical elements on the detection path were antireflection coated for the visible region (Thorlabs, A). The water immersion objective lenses had the following specifications: The bottom objective lens (Olympus, LUMFLN 60XW) had a 60x, NA 1.1, 1.5 mm working distance, and a collar for variable coverslip correction. The top objective lens (Olympus, LUMPFLN 60XW) had a 60x, NA 1.0, 2 mm working distance, and no collar for variable coverslip correction. The tube lenses were provided by the manufacturer of the objective lenses (Olympus, U-TLU). The two incident beams were generated with an SLM (Holoeye, Pluto-NIR-015) displaying a diffraction grating. The sample was mounted on an XYZ translation stage (AsiImaging, PZ-2000). The XY-axes were controlled by actuators and had 5 cm travel range, whereas the Z-axis consisted of a piezoelectric stage with 300 µm travel range. The objective lenses were mounted on single axis actuator stages (Asi Imaging, LS-200). All the stages were mounted on a modular system (Asi Imaging, RAMM). SHG signal was obtained by filtering the detected light with a 515±10 nm bandpass filter (FL514.5-10, Thorlabs). The residual excitation light was removed with a 910 nm shortpass filter (ET910SP, Chroma). The imaging camera was a back-illuminated electron-multiplied and intensified CCD camera with 512x512 pixels (PI-MAX4: 512EM-HBf P46 GEN III). The incident polarization was controlled with a quarter-waveplate (WPQ05M-1030, Thorlabs) followed by a half-waveplate (WPH05M-1030, Thorlabs). The emitted SHG was detected for a polarization either X or Y axis with a rotating half-wave plate (WPH10M-514, Thorlabs) followed by a polarizing beam splitter (Thorlabs). Images were acquired using 10x 0.25 s acquisition time.

Second Harmonic Spectroscopy (SHS)

Second harmonic scattering measurements were performed on the same SHS setup as described previously.[2] Briefly, 190 fs laser pulses at a center wavelength of 1030 nm, with a repetition rate of 200 kHz and average power of 60 mW (laser: Pharos SP, LightConversion), were focused into a cylindrical glass sample cell (4.2 mm inner diameter, high precision cylindrical glass cuvettes, LS instruments). The input- polarization was controlled by a Glan-Taylor polarizer (GT10-B, Thorlabs) and a zero-order half-wave plate (WPH05M-1030, Thorlabs), while the output-polarization was controlled with another Glan Taylor polarizer (GT10-A, Thorlabs). A long pass filter (FEL0750, Thorlabs) cleaned the fundamental beam of other frequencies before the beam was focused. The beam waist at focus on the sample was about ~35 µm; the corresponding Rayleigh length was ~0.94 mm. The scattered SHG light was

collected, collimated with a plano-convex lens (f=5 cm), polarization analyzed and filtered (515±10 nm bandpass filter, Chroma) before being focused into a gated photomultiplier tube (H7421-40, Hamamatsu). The acceptance angle was set to 3.4° for scattering patterns. Patterns were obtained in steps of 5° from -90° to 90° with 0° being the forward direction of the fundamental. Data points were acquired using 30x 1 second acquisition time with a gate width of 10 nanoseconds.

Symmetry of the bioharmonophore content

The SHG response of a medium is characterized by its second order nonlinear optical susceptibility tensor $\chi^{(2)}$, which connects the induced polarization $P^{2\omega}$ to the incident electric field E^{ω}:[3]

$$P_I = \sum \chi_{IJK} E_J E_K$$

where I, J and K stand for X, Y or Z spatial directions in the laboratory frame. The SHG response of the bioharmonophores therefore depended on the second order susceptibility tensor $\chi^{(2)}$. This tensor $\chi^{(2)}$ has different non-zero components depending on the symmetries of the assembly of molecules inside the nanoparticles.

Here, we expected two possible organizations of the FFF peptides inside the bioharmonophores[4]:

either they are organized in a crystalline phase with a orthorhombic D_2 symmetry;

or in a monoclinic C_2 symmetry.

In the first case, the non-zero components in the tensor $\chi^{(2)}$ are:

$\chi^{(2)}_{xyz} = \chi^{(2)}_{xzy}$, $\chi^{(2)}_{yxz} = \chi^{(2)}_{yzx}$ and $\chi^{(2)}_{zxy} = \chi^{(2)}_{zyx}$

So, for an incident polarization $\vec{E}^{2\omega} = \begin{bmatrix} \cos(\alpha) \\ \sin(\alpha)\cos(\delta) \\ \pm\sin(\alpha)\sin(\delta) \end{bmatrix}$, with 2δ the angle between the two incident beams and α the incident polarization angle with respect to X axis, we expected the following trend for the SHG intensity detected either along X or Y axes:

$$I_X = |P_X|^2 = A_X \sin^4(\alpha - \varphi)$$
$$I_Y = A_Y \sin^2(2\alpha - 2\varphi)$$

The experimental polarimetric diagrams did not follow this trend.

In the second case, the non-zero components in the tensor $\chi^{(2)}$ are:

$\chi^{(2)}_{xxx}$, $\chi^{(2)}_{xzz}$, $\chi^{(2)}_{xyy}$, $\chi^{(2)}_{zxz} = \chi^{(2)}_{zzx}$, $\chi^{(2)}_{yxy} = \chi^{(2)}_{yyx}$, $\chi^{(2)}_{yzx} = \chi^{(2)}_{yxz}$ and $\chi^{(2)}_{zxy} = \chi^{(2)}_{zyx}$

For the simplest case of a bioharmonophore with its axis in the (XY) plane (image plane of the microscope), if we suppose the excitation beams are propagating along Z and under the Kleinman symmetry (all indices can permute), the expected SHG intensity is

$$I_X(\alpha) = A_X \cos(4\alpha - 4\varphi) + B_X \cos(2\alpha - 2\varphi) + C_X$$
$$I_Y(\alpha) = A_Y \sin(2\alpha - 2\varphi)^2$$

with α the angle of the incident polarization with respect to X axis and φ the angle of the particle's axis with respect to X.

The same equations rule the SHG signal if the excitation beams have an incident angle δ, if there is a bit of disorder or if the axis is not in the image plane, as long as there is a symmetry

axis. In that case the coefficients A_X, B_X, C_X and A_Y are different. Those equations fit well the data we obtained for the 4 different bioharmonophores observed, allowing us to suppose the nanoparticles display here a cylindrical symmetry. The patterns for those 4 bioharmonophores have the same shape but are slightly different, corroborating the fact that bioharmonophores have a particular axis that is oriented differently for each probe.

Supplementary Note 2 - Determining the fraction of bioharmonophore-labeled tumors in a zebrafish cancer model.

This section outlines the experimental design and strategies (i) to ascertain SHG signal specificity in zebrafish xenograft models and (ii) to quantify tumor labeling efficiency of bioharmonophores with different surface coatings.

<u>*Zebrafish Embryo Experimental Group Design*</u>

We divided zebrafish embryos into 6 separate experimental groups (group A-F) that comprised of embryos that were not injected (group A), injected only with bioharmonophores (group B and C), injected only with cancer cells (group D), and injected with cancer cells followed by bioharmonophore (groups E and F).

The first zebrafish group (group A) consisted of 5 days post fertilization (dpf) embryos that were not injected with bioharmonophores or cancer cells. This group acted as a negative control to assess the presence of background SHG signal that would interfere with the quantification of tumor bioharmonophore labeling efficiency (see below *Zebrafish Embryo Imaging* and *SHG Signal Background*).

The second and third zebrafish group (group B and C) embryos were injected with PEG-coated and p32 targeting peptide-coated bioharmonophores, respectively (see below *Bioharmonophore administration*). Both groups were used to follow biodistribution and clearance of bioharmonophores with different coatings (see below *Bioharmonophore Biodistribution and Clearance*).

The fourth zebrafish group (group D) consisted of xenograft embryos that were injected with DsRed-expressing metastatic human melanoma cells. This group was used to evaluate whether tumor regions in zebrafish models develop SHG background signal that could lead to false-positives readouts (see below *Zebrafish Xenograft Cancer Model*).

The fifth and sixth zebrafish group (group E and F) contained xenograft embryos that were injected with cancer cells at 2 dpf and PEG-coated (group E) and p32 targeting peptide-coated (group F) bioharmonophores at 3 dpf. These groups were used to determine the specificity and the labeling efficiency of targeted bioharmonophores for *in Vivo* imaging applications (see below *Statistical Evaluation of Tumor Labeling Efficiency using Targeted Bioharmonophores*).

<u>*Zebrafish Embryo Imaging*</u>

For all zebrafish groups (A-F), entire zebrafish embryos were imaged with a Zeiss LSM 780 Confocal Microscope. The 488 nm laser was used to visualize GFP labeled endothelial cells, the 561 nm laser was used to localize dsRed expressing cancer cells, and the 850 nm near-infrared (NIR) laser was used to visualize background or bioharmonophore SHG signal. In the case of groups D-F that contained cancer cells, SHG imaging was performed at each tumor site.

SHG Signal Background

Obtained results showed that zebrafish embryos (group A) did not produce SHG background signal with the exception of minimal endogenous SHG signal originating from a large and elongated structure in the zebrafish tail. This tail region was excluded from assessing specificity of tumor targeting (**Supplementary Figure 19**).

Bioharmonophore administration

Bioharmonophores were injected at 3 dpf into the Duct of Cuvier (DoC) region and subsequently imaged at 5 dpf. Given the small diameter of the injection needle required for injection into the DoC region, we administered a limited number of bioharmonophores (10 nl of stock solution with 6 mg/ml triphenylalanine concentration) to prevent clogging.

Bioharmonophore Biodistribution and Clearance

In groups B and C, bioharmonophores were present in various parts of injected zebrafish embryos and did not aggregate at the injection site (**Supplementary Figure 17d,g**). Bioharmonophores tended to be cleared out of circulation 24 hours after injection, possibly via macrophages and scavenger endothelial cells,[5,6] as evidenced by the absence of SHG signal (**Supplementary Figure 17e,h**).

Zebrafish Xenograft Cancer Model

To generate a statistically significant number of tumors for each zebrafish embryo (group D), we employed the highly aggressive MDA-MB-435-DsRed cancer cell line that can metastasize to multiple locations, forming distinct tumor regions within one day.[7] As outlined in previous studies,[8] we injected 200-300 cancer cells at 2 dpf to yield a tumor distribution sample size that offers sufficient statistical power at 5 dpf, without causing premature death **(Supplementary Figure 18)**. Tumor sizes varied highly within entire embryos that were investigated, ranging from single cancer cells to tens of cancer cells. To maintain an unbiased approach that would take into account these variations, we defined a distinct tumor site as a cell or group of cells that is separated by at least 60 microns (3-4 cell width) from another tumor.[9] The number of tumors in the xenograft zebrafish groups D, E and F did not show a difference that is statistically significant **(Supplementary Figure 20)**. Importantly, the tumor sites in group D did not generate any SHG signal **(Supplementary Figure 18a-c)**, indicating that any SHG signal detected at a tumor site originated from targeted bioharmonophores (see above *Zebrafish Embryo Imaging*).

Statistical Evaluation of Tumor Labeling Efficiency using Targeted Bioharmonophores

At 5 dpf, embryos of groups E and F were imaged to assess colocalization between cancer cells and bioharmonophore signal (see above *Zebrafish Embryo Imaging*). The 48 hour delay

between bioharmonophore administration and zebrafish embryo imaging ensured that unbound bioharmonophores (i.e. false-positive colocalizing events) were removed from the embryo, while cancer cells continued to divide and to metastasize to different regions.

Not all cancer cells of a tumor tended to be labeled **(Figure 4c)**. This observation is potentially due to limited blood vessel leakiness and restricted accessibility to tightly packed cancer cells within tumors, limiting the extravasation of bioharmonophores and their access to p32 receptors. In addition, cancer cells continued to proliferate and spread throughout the zebrafish embryo between single bioharmonophore administration and zebrafish embryo imaging.

To account for this finding, we decided to define a labeled tumor as a site where at least a single cancer cell colocalized with SHG signal that originated from targeted bioharmonophores (see above *Zebrafish Embryo Imaging*). Since tumor numbers and volumes varied between individual zebrafish xenografts, labeling efficiency was defined as the ratio of bioharmonophore-labeled tumors to the total amount of tumors in the whole zebrafish embryo (**Figure 4d**). To account for the high variation of tumor numbers within zebrafish groups and to ascertain statistical significance of acquired results, a non-parametric Kruskal-Wallis test with Dunn's post hoc multiple comparison was applied. The zebrafish cancer model injected with p32 peptide-targeted bioharmonophores had a significantly higher fraction of labeled tumors compared with non-injected and PEG-coated bioharmonophores (**Figure 4d**).

Supplementary References

1. Macias-Romero, C.; Nahalka, I.; Okur, H. I.; Roke, S., Optical Imaging of Surface Chemistry and Dynamics in Confinement. *Science* **2017,** *357* (6353), 784-788.

2. Gomopoulos, N.; Lutgebaucks, C.; Sun, Q.; Macias-Romero, C.; Roke, S., Label-Free Second Harmonic and Hyper Rayleigh Scattering with High Efficiency. *Opt Express* **2013,** *21* (1), 815-21.

3. Boyd, Robert W., Nonlinear Optics, Third Edition. Academic Press/Elsevier. London. 2008; p 640.

4. Handelman, A.; Lavrov, S.; Kudryavtsev, A.; Khatchatouriants, A.; Rosenberg, Y.; Mishina, E.; Rosenman, G., Nonlinear Optical Bioinspired Peptide Nanostructures. *Advanced Optical Materials* **2013,** *1* (11), 875-884.

5. Evensen, L.; Johansen, P. L.; Koster, G.; Zhu, K.; Herfindal, L.; Speth, M.; Fenaroli, F.; Hildahl, J.; Bagherifam, S.; Tulotta, C.; Prasmickaite, L.; Maelandsmo, G. M.; Snaar-Jagalska, E.; Griffiths, G., Zebrafish as a Model System for Characterization of Nanoparticles against Cancer. *Nanoscale* **2016,** *8* (2), 862-877.

6. Campbell, F.; Bos, F. L.; Sieber, S.; Arias-Alpizar, G.; Koch, B. E.; Huwyler, J.; Kros, A.; Bussmann, J., Directing Nanoparticle Biodistribution through Evasion and Exploitation of Stab2-Dependent Nanoparticle Uptake. *Acs Nano* **2018,** *12* (3), 2138-2150.

7. Marques, I. J.; Weiss, F. U.; Vlecken, D. H.; Nitsche, C.; Bakkers, J.; Lagendijk, A. K.; Partecke, L. I.; Heidecke, C.-D.; Lerch, M. M.; Bagowski, C. P., Metastatic Behaviour of Primary Human Tumors in a Zebrafish Xenotransplantation Model. *BMC Cancer* **2009,** *9* (1), 43.

8. Konantz, M.; Balci, T. B.; Hartwig, U. F.; Dellaire, G.; André, M. C.; Berman, J. N.; Lengerke, C., Zebrafish Xenografts as a Tool for *in Vivo* Studies on Human Cancer. *Annals of the New York Academy of Sciences* **2012,** *1266* (1), 124-137.

9. Lee, S. L.; Rouhi, P.; Dahl Jensen, L.; Zhang, D.; Ji, H.; Hauptmann, G.; Ingham, P.; Cao, Y., Hypoxia-Induced Pathological Angiogenesis Mediates Tumor Cell Dissemination, Invasion, and Metastasis in a Zebrafish Tumor Model. *Proc Natl Acad Sci U S A* **2009,** *106* (46), 19485-90.
